# Cystargamides C and D, New Cyclic Lipopeptides From a Tidal Mudflat-Derived *Streptomyces* sp. JMS132

**DOI:** 10.3389/fmicb.2022.904954

**Published:** 2022-05-13

**Authors:** Jeongwon Seo, Yern-Hyerk Shin, Se Jin Jo, Young Eun Du, Soohyun Um, Young Ran Kim, Kyuho Moon

**Affiliations:** ^1^College of Pharmacy, Research Institute of Pharmaceutical Sciences, Chonnam National University, Gwangju, South Korea; ^2^Department of Biological Chemistry and Molecular Pharmacology, Harvard Medical School and Blavatnik Institute, Boston, MA, United States; ^3^Natural Products Research Institute, College of Pharmacy, Seoul National University, Seoul, South Korea; ^4^College of Pharmacy, Yonsei University, Incheon, South Korea

**Keywords:** tidal mudflat, lipodepsipeptide, cystargamide, structural determination, bacterial secondary metabolite, antioxidant activity

## Abstract

Cystargamides C and D (**2** and **3**) were isolated from a marine actinomycete strain collected at Beolgyo, South Korea. The planar structures of the cystargamides were elucidated by 1/2D NMR, UV, and MS spectroscopic analyses. The absolute configurations of **2** and **3** were determined based on ROESY correlations and the advanced Marfey’s methods. The structures of the compounds were elucidated as new lipodepsipeptides bearing six amino acids with an epoxy fatty acid side chain. For the first time, the nonribosomal peptide synthetase biosynthetic pathway of the cystargamides has been proposed using whole genome sequence analysis. The cystargamides displayed antioxidant effect in the DPPH and ABTS assay. The discovery of new cyclic lipopeptides, cystargamides C and D, from a tidal mudflat-derived *Streptomyces* sp. supported that marine bacteria have potential as source of bioactive natural products.

## Graphical Abstract

*Streptomyces* sp. J MS1 32. 
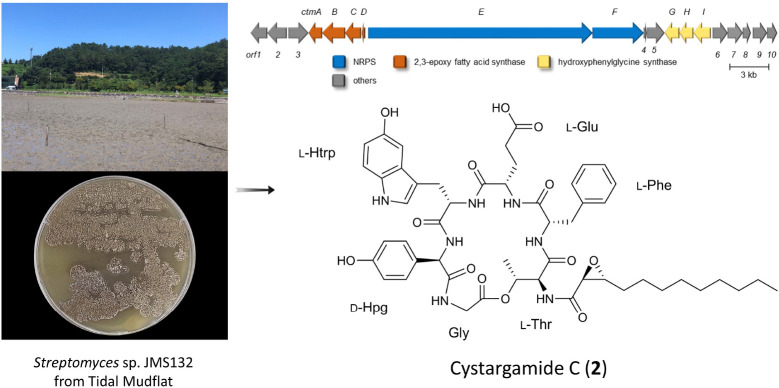

## Introduction

The ocean accounts for 70% of the Earth’s surface area and consists of diverse environments including sedimentary layers such as tidal flats. Bacteria living in tidal flats where high and low tides are repeated, and with extreme changes in salinity, water pressure, temperature, and sunlight are adapted to severe environmental changes. Additionally, they are expected to be unreported specificity, which means that unique marine environments-derived bacteria have undiscovered biosynthetic gene clusters (BGCs) to produce new secondary metabolites (Choi et al., 2018; Kim et al., 2018; Moon et al., 2019a,b; Bae et al., 2020; Du et al., 2021). In particular, the genus *Streptomyces* accounted for 62.5% of the reported natural products derived from marine bacteria until 2019 (Carroll et al., 2021). Previously, secondary metabolites derived from tidal mud flat actinomycetes such as boholamide A (Seo and Moon, 2021), WS9326H, mohangic acids A–E, hormaomycins B and C, mohangamides A and B (Bae et al., 2015a,b, 2016, 2018), and buanmycin and buanquinone (Moon et al., 2015) have been studied. As part of our continuous efforts to discover new bioactive natural products from microorganisms, we isolated actinomycete strains from the tidal mud flat in Beolgyo, South Korea, and studied the bacterial chemical profiles of their secondary metabolites using liquid chromatography–mass spectrometry (LC/MS). Based on ultraviolet (UV) and MS data, the *Streptomyces* sp. JMS132 strain produced peptides containing an indole moiety (UV λ_max_ = 275 nm, [M+H]^+^ *m/z* at 982 and 926). From a large-scale cultivation of the strain and purification, two new cyclic lipodepsipeptide cystargamides C (**2**) and D (**3**) were isolated with previously known cystargamide B (**1**) (Kitani et al., 2018). Cystargamide B bears threonine, phenylalanine, glutamic acid, 5-hydroxytryptophan, 4-hydroxyphenylglycine, and glycine as well as a trans-2,3-decanoyl acid in its structure, and has a different configuration of 4-hydroxy phenylglycine in cystargamide A (Gill et al., 2014). In spite of the presences of biosynthetically unique features in the cystargamides, such as 4-hydroyxphenylglycine and trans-2,3-epoxy fatty acid, their biosynthetic gene cluster has not been studied in previous studies, which led us to investigate on their biosynthetic pathway. In the case of their bioactivities, cystargamide B was reported to inhibit NS2B/NS3 complex protease from the dengue virus.

In our study, the planar structures of the cystargamides C and D were elucidated using 1D and 2D nuclear magnetic resonance (NMR), mass, UV, and infrared (IR) spectroscopic analysis. The absolute configurations of the compounds were determined by the advanced Marfey’s method, ROESY analysis, and biosynthetic gene clusters analysis. Furthermore, we suggested a biosynthesis mechanism of cystargamides based on microbial whole genome sequencing and reported biological evaluation of **1–3**. These results showed significance of marine microbial-derived unreported molecules and their potential medical applications.

## Materials and Methods

### General Experimental Procedures

Optical rotations were measured using a polarimeter (Model 343 plusPerkinElmer, MA, United States). IR spectra were acquired using a spectrometer (Spectrum 3, PerkinElmer, MA, United States). UV spectra and LC/MS data were recorded using an Agilent G6125B MSD system coupled with an Agilent Technologies 1260 series Infinity II LC system using a reversed-phase C_18_ column (Phenomenex Luna, 100 × 4.6 mm, 5 μm). Circular dichroism (CD) spectra were recorded on a Chirascan V100 CD spectrometer (Applied Photophysics Ltd., United Kingdom) using a 1 mm CD cell. High-resolution electrospray ionization mass spectra (HR-ESI-MS) were obtained using an Agilent Technologies 1290 series HPLC coupled with an Agilent 6530 iFunnel Q-TOF LC/MS system. ^1^H, ^13^C, and 2D NMR spectra were recorded on a Bruker Avance II 900 MHz and a Bruker Advance III 700 MHz spectrometer at the Korea Basic Science Institute in Ochang. HPLC purification was performed on a Waters system (1525 binary HPLC pump and 996 photodiode array detector) with a YMC Pack-ODS-A-C_18_ column (250 × 10 mm, 5 μm).

### Collection of Tidal Flat Samples and Isolation of Bacterial Strain

The tidal flat sediment samples were collected at Beolgyo, South Korea in August 2020. Mixtures of dried sediment samples and 4 mL of sterilized water (2 g each) were heated at 55°C and sonicated. The mixtures were spread on actinomycete isolation medium, A1 medium, B1 medium, starch casein medium, chitin-based medium, Kuster medium, and TWYE medium with cycloheximide (25 mg/L) and gentamycin (5 mg/L) and incubated at 25°C for 20 days. The JMS132 strain was isolated from M1 medium and identified as *Streptomyces* sp. (98.8% identical to *Streptomyces malachitofuscus*) based on the 16S rRNA gene sequence analysis (GenBank accession number AB184282).

### Large-Scale Cultivation and Extraction

The JMS132 strain was grown in 50 mL of bennet medium (glucose 10 g, yeast extract 1 g, beef extract 1 g, tryptone 2 g, and 1 L of seawater) in a 100 mL flask and incubated at 25°C for 2 days on a rotary shaker at 190 rpm. A 3.5 mL aliquot of the broth culture was used to inoculate 150 mL of bennet medium in a 500 mL flask for initial scale-up and incubated at the same conditions. For the large-scale cultivation, 20 mL of the culture was transferred to 1 L of bennet medium in 2.5 L ultra-yield flasks (8 ea × 1 L, total volume 8 L) for 3 days at the same fermentation conditions. A whole culture of the JMS132 strain was extracted with 14 L of ethyl acetate (EtOAc). The EtOAc and water layers were separated and the remaining water in the EtOAc layer was removed by adding anhydrous sodium sulfate. The EtOAc extract was concentrated *in vacuo* to yield 873 mg of the extract.

### Isolation and Purification of Cystargamides (1–3)

The JMS132 extract was fractionated over a C_18_ reversed-phase column (YMC ODS-A C_18_, 50 μm silica gel) using a step gradient solvent system (20, 40, 60, 80, 100% MeOH/H_2_O and 1:1 MeOH/DCM). After the fractionation, each fraction was monitored by LC/MS analysis, and cystargamide B (**1**), cystargamide C (**2**) were detected in the 80% and 100% of the MeOH fractions, and cystargamide D (**3**) in the 60% MeOH fraction. Each fraction was filtered with a syringe filter (Advantec, HP020AN) and then subjected to semi-preparative reversed-phase HPLC with a flow rate of 2 mL/min using a linear gradient from 30 to 90% CH_3_CN/H_2_O for 40 min (YMC ODS-A-C_18_: 250 × 10 mm, 5 μm, UV detection at 280 nm). Under these HPLC conditions, compounds **1**, **2**, and **3** were eluted at 26, 29, and 11 min, respectively. Finally, the samples were further purified to yield pure **1** (24 mg), **2** (22 mg), and **3** (6 mg).

#### Cystargamide B (**1**)

Brownish oil; [α]^20^_D_ +7.3 (c 0.055, MeOH); IR (neat) ν_max_ 3302, 2927, 1654, 1516, 1204 cm^–1^; UV (MeOH) λ_max_ (log ε) 275 (5.71) 300 (5.51) nm; ^1^H and ^13^C NMR data (see Supplementary Table 1); HR-ESI-MS [M + H]^+^ *m/z* 954.4228 (calcd. for C_49_H_59_N_7_O_13_, 954.4249, error: –2.2 ppm).

#### Cystargamide C (**2**)

Brownish oil; [α]^20^_D_ +3.8 (c 0.055, MeOH); IR (neat) ν_max_ 3287, 2925, 1656, 1515, 1203 cm^–1^; UV (MeOH) λ_max_ (log ε) 275 (5.43) 300 (5.22) nm; ^1^H and ^13^C NMR data (see Table 1); HR-ESI-MS [M + H]^+^ *m/z* 982.4541 (calcd. for C_51_H_63_N_7_O_13_, 982.4562, error: –2.1 ppm).

**TABLE 1:** NMR spectral data for cystargamide C and D (**2–3**) in DMSO-*d*_6_.

Position		2*^a^*		3*^b^*	
		δ_C_, type	δ_H_, mult (J, Hz)	δ_C_, type	δ_H_, mult (J, Hz)
Epd	CO	167.8, C		167.8, C	
	2	53.6, CH	3.42, d (1.5)	53.6, CH	3.42, d (2.0)
	3	57.7, CH	2.81, dt (4.5, 1.5)	57.7, CH	2.81, dt (4.5, 2.0)
	4	30.7, CH_2_	1.56, m; 1.48, m	30.7, CH_2_	1.56, m; 1.48, m
	5^c^	25.2, CH_2_	1.36, m; 1.28, m	24.9, CH_2_	1.37, m; 1.28, m
	6^c^	28.7, CH_2_	1.36, m; 1.25, m	30.8, CH_2_	1.28, m
	7^c^	28.9, CH_2_	1.25, m	22.0, CH_2_	1.29, m
	8^c^	28.9, CH_2_	1.25, m	13.9, CH_3_	0.87, t (7.0)
	9^c^	28.7, CH_2_	1.25, m		
	10^c^	31.3, CH_2_	1.24, m		
	11^c^	22.1, CH_2_	1.25, m		
	12	14.0, CH_3_	0.85, t (7.0)		
Thr	CO	168.5, C		168.2, C	
	NH		7.69, d (9.0)		7.69, d (9.0)
	α	54.1, CH	4.54, d (9.0)	54.2, CH	4.54, d (9.0)
	β	70.3, CH	5.37, m	70.4, CH	5.37, m
	γ	16.3, CH_3_	1.10, d (6.0)	16.3, CH_3_	1.09, d (6.0)
Phe	CO	170.1, C		170.2, C	
	NH		8.64, m		8.72, m
	α	54.2, CH	4.45, m	54.2, CH	4.45, m
	β	36.9, CH_2_	3.10, m; 2.70, m	36.9, CH_2_	3.10, m; 2.70, m
	1′	138.0, C		138.0, C	
	2′/6′	129.0, CH	7.16, m	129.0, CH	7.16, m
	3′/5′	128.0, CH	7.19, m	128.0, CH	7.19, m
	4′	126.1, CH	7.13, m	126.1, CH	7.13, m
Glu	CO	171.8, C		171.7, C	
	NH		8.22, m		8.22, m
	α	52.1, CH	4.28, m	51.9, CH	4.28, m
	β	28.7, CH_2_	2.00, m; 1.89, m	28.6, CH_2_	2.00, m; 1.89, m
	γ	32.9, CH_2_	2.37, m; 2.23, m	32.4, CH_2_	2.36, m; 2.22, m
	COOH	176.1, C		175.6, C	
Htrp	CO	171.6, C		171.6, C	
	NH		8.22, m		8.22, m
	α	55.2, CH	4.33, m	55.2, CH	4.33, m
	β	26.2, CH_2_	2.95, m	26.2, CH_2_	2.95, m
	1		10.52, m		10.53, m
	2	124.3, CH	7.06, m	124.2, CH	7.05, m
	3	108.3, C		108.3, C	
	3a	127.9, C		127.9, C	
	4	102.3, CH	6.86, m	102.3, CH	6.85, m
	5	150.4, C		150.4, C	
	6	111.3, CH	6.54, m	111.3, CH	6.55, m
	7	111.5, CH	7.05, m	111.5, CH	7.05, m
	7a	130.6, C		130.6, C	
Hpg	CO	170.3, C		170.4, C	
	NH		8.70, m		8.72, m
	α	56.0, CH	5.23, m	56.0, CH	5.21, m
	1′	127.9, C		128.0, C	
	2′/6′	129.4, CH	6.88, m	129.3, CH	6.87, m
	3′/5′	114.7, CH	6.60, m	114.8, CH	6.60, m
	4′	156.6, C		156.7, C	
Gly	CO	168.5, C		168.5, C	
	NH		8.22, m		8.22, m
	α	40.4, CH_2_	4.31, m; 3.54, m	40.4, CH_2_	4.31, m; 3.55, m

*^a1^H and ^13^C data were recorded at 900 and 225 MHz, respectively.*

*^b1^H and ^13^C data were recorded at 700 and 175 MHz, respectively.*

*^c^Overlapped signals.*

#### Cystargamide D (**3**)

Brownish oil; [α]^20^_D_ +9.3 (c 0.018, MeOH); IR (neat) ν_max_ 3288, 2926, 1654, 1516, 1209 cm^–1^; UV(MeOH) λ_max_ (log ε) 275 (5.51) 300 (5.31) nm; ^1^H and ^13^C NMR data (see Table 1); HR-ESI-MS [M + H]^+^ *m/z* 926.3972 (calcd. for C_47_H_55_N_7_O_13_, 926.3936, error: +3.9 ppm).

### Oxidation and Acid Hydrolysis of Cystargamides (1–3)

Each 3 mg of cystargamides was added 2.6 mg of NaIO_4_ and dissolved in a mixture of 1 mL CHCl_3_. 45 mg of RuCl_3_⋅nH_2_O was diluted with 6 mL CH_3_CN and 9 mL H_2_O. The solution was mixed with the samples and then 1 mL of 6 mL CH_3_CN and 9 mL H_2_O solution was added to each sample. After the reaction mixtures were stirred for 2 h, the mixtures were added 10 mL H_2_O and extracted with 20 mL CH_2_Cl_2_ and 30 mL EtOAc. The samples were concentrated *in vacuo* and dissolved in 6 N HCl (1 mL) and then heated at 90°C for 17 h. The hydrolysate was immediately cooled at 0°C for 5 min and concentrated *in vacuo*. To remove the residual HCl in the reaction mixture, 1 mL of distilled water was added to the vial and evaporated under low pressure; this process was repeated three times.

### Determination of the Absolute Configurations by Advanced Marfey’s Method

The absolute configurations of the cystargamides were determined using LC/MS based advanced Marfey’s method (Harada et al., 1996). The hydrolysate was lyophilized for 24 h and divided into two separate vials. 1 N NaHCO_3_ (500 μL) and 10 mg/mL 1-fluoro-2,4-dinitrophenyl-5-L-leucine amide (L-FDLA) in acetone (100 μL) solutions were added to one vial, and the reaction mixtures were stirred at 80°C for 12 min and then neutralized with 100 μL of 2 N HCl. In the same way, the D-FDLA derivatives were also prepared. The L- and D-FDLA products were diluted with 1:1 CH_3_CN/H_2_O and then analyzed using LC/MS on a reversed-phase column (Phenomenex C_18_: 100 × 4.6 mm, 5 μm, CH_3_CN/water = 20/80 to 60/40, 50 min with 0.1% formic acid, flow rate = 0.4 mL/min). The retention times of the L-/D-FDLA derivative of the amino acid units were identified using UV chromatogram figure and MS ion extraction. L-/D-FDLA derivatized amino acids as a standard were prepared by reacting with 0.5 mg of authentic amino acids in the same way as described above. To determine the absolute configurations of the amino acids, the retention times were compared with the authentic derivatives; the standard amino acids eluted at 25.2 min (L-Thr), 38.2 min (L-Phe), 27.7 min (L-Glu), 32.2 min (L-Hpg), and 26.7 min (L-Asp) (Supplementary Figures 25, 26 and Supplementary Table 2).

### Genome Sequencing and Gene Annotation of the *Streptomyces* sp. JMS132

Full genome sequence data of the cystargamides-producing strain, *Streptomyces* sp. JMS132, were acquired by Macrogen, Inc. using PacBio RSII Sequencer and Illumina HiSeqXten sequencer. The genomic sequence data were assembled and resulted in 7 715 443 bp (1 contig) of sequence data. The result of annotation revealed that the *Streptomyces* sp. JMS132 genome was composed of 72.5% G+C, 6807 coding DNA sequences (CDSs), and 86 tRNA genes. The antiSMASH 6.0 software program was used to determine biosynthetic gene clusters of cystargamides (Blin et al., 2021).

### DPPH Free-Radical Scavenging Assay

The free-radical scavenging activity was measured using the 2,2-diphenyl-1-picrylhydrazyl (DPPH) assay (Park et al., 2016). Vitamin C was used as a positive antioxidant drug. Cystargamides (50, 100, and 200 μg/mL) and vitamin C (10 μg/mL) dissolved in methanol were mixed with 0.4 mM of methanolic DPPH at room temperature for 30 min. The absorbance was read at 490 nm using an ELISA microplate reader (ELx808) (BioTek Instruments, Inc., VT, United States).

### ABTS Radical Scavenging Assay

The cation-radical scavenging activity was measured by the 2,2-azino-bis (3-ethylbenzothiazoline-6-sulfonic acid) (ABTS) assay (Ilyasov et al., 2020). Methanol solution with 7.4 mM ABTS and 2.45 mM potassium persulfate was stored in the dark at room temperature for approximately 16–24 h before use. Vitamin C was used as a positive antioxidant drug. Cystargamides (50, 100, and 200 μg/mL) and vitamin C (10 μg/mL) dissolved in methanol were mixed with methanolic ABTS cation radical at room temperature for 30 min. The absorbance was read at 750 nm using an ELISA microplate reader.

The scavenging percentage of DPPH or ABTS was calculated according to the following equation:


$$\text{Scavenging}\left(\%\right)=\frac{A\,b\,s\,o\,r\,b\,a\,n\,c\,e\,o\,f\,c\,o\,n\,t\,r\,o\,l-A\,b\,s\,o\,r\,b\,a\,n\,c\,e\,o\,f\,t\,h\,e\,s\,a\,m\,p\,l\,e}{A\,b\,s\,o\,r\,b\,a\,n\,c\,e\,o\,f\,c\,o\,n\,t\,r\,o\,l}\times 100$$

### Statistical Analysis

The DPPH and ABTS assay results were expressed as mean ± standard error of the mean (SEM) unless otherwise stated. Statistical differences were evaluated using one-way ANOVA for multi-group comparisons followed by a Tukey *post-hoc* test, and a *p*-value < 0.05 considered significant.

## Results and Discussion

### Identification and Structure Elucidation of Cystargamides From *Streptomyces* sp. JMS132

**FIGURE 1:**
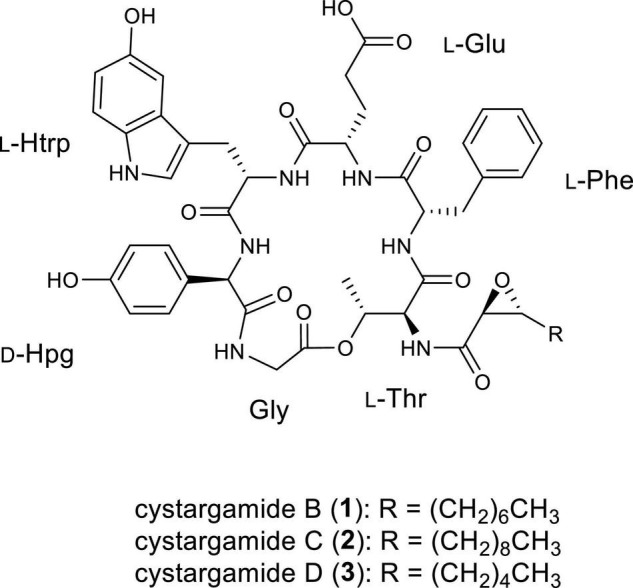
Structures of cystargamides B, C, and D (**1–3**).

Cystargamide B (**1**) was isolated as a brownish oil. The UV absorption peak at 275 nm suggested that **1** is a peptide with indole. The molecular formula was deduced as C_49_H_59_N_7_O_13_, which has 24 degrees of unsaturation, based on its HR-ESI mass spectrometric data and ^1^H and ^13^C NMR data. The structure of **1** is identical to that of cystargamide B (Kitani et al., 2018; Figure 1).

Cystargamide C (**2**) was isolated as a brownish oil, its UV spectra was identical to that of **1** and its ^1^H NMR chemical shifts were very similar to those of **1** (Table 1). The molecular formula of **2** was determined to be C_51_H_63_N_7_O_13_ based on its HR-ESI mass spectrometric data and ^1^H and ^13^C NMR data. The ^1^H and HSQC NMR spectra data of **2** indicated the presence of seven exchangeable protons [δ_H_ 10.52, 8.70, 8.64, 8.22 (3H), and 7.69] and ten heteroatom-bound methine protons (δ_H_ 5.37, 5.23, 4.54, 4.45, 4.33, 4.31, 4.28, 3.54, 3.42, and 2.81). Detailed analysis of these data showed the existence of 13 olefinic protons [δ_H_ 7.19 (2H), 7.16 (2H), 7.13, 7.06, 7.05, 6.88 (2H), 6.86, 6.60 (2H), and 6.54], 12 aliphatic methylene groups (δ_H_ 3.10–1.24), and 2 methyl groups (δ_H_ 1.10 and 0.85). The ^13^C NMR data also revealed eight carbonyl carbons [δ_C_ 176.1, 171.8, 171.6, 170.3, 170.1, 168.5 (2C), and 167.8] and nine heteroatom-bound carbons (δ_C_ 70.3, 57.7, 56.0, 55.2, 54.2, 54.1, 53.6, 52.1, and 40.4), which indicated the structural features of peptide-derived compound. This data also indicated 14 aliphatic sp^3^ carbons including two methyl groups (δ_C_ 36.9–14.0).

A comprehensive analysis of the 2D NMR data of **2** amino acid side chains were assigned using ^1^H, ^13^C, COSY, TOCSY, HSQC, HMBC, and ROESY spectra. The COSY and HMBC NMR spectra revealed structures of four proteinogenic amino acids (threonine [Thr], phenylalanine [Phe], glutamic acid [Glu], and glycine [Gly]) and two non-proteinogenic amino acids (5-hydroxy tryptophan and, *p*-hydroxy phenylglycine).

**FIGURE 2:**
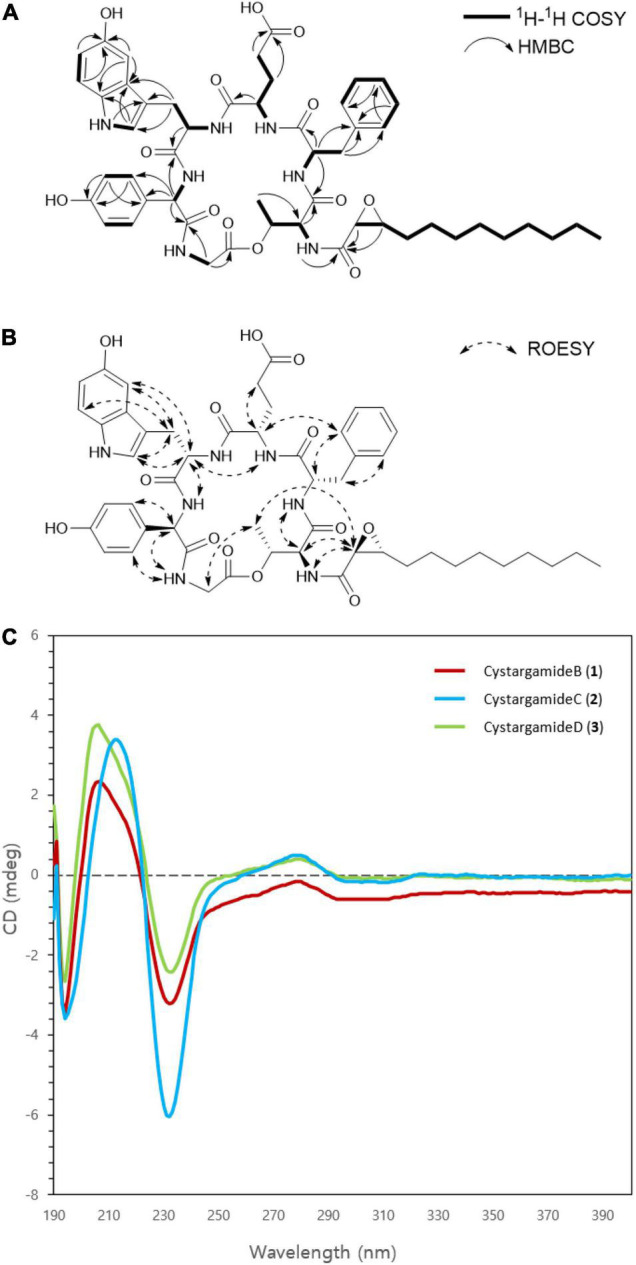
**(A)** Key COSY and HMBC correlations in cystargamide C (**2**). **(B)** Key ROESY correlations in cystargamide C (**2**). **(C)** The circular dichroism (CD) spectra of cystargamides B–D (**1–3**) at a concentration of 0.25 mg/mL in MeOH.

The four proteinogenic amino acids, Thr, Phe, Glu, and Gly, were assigned based on the chemical shift values and 2D NMR data. One of the non-proteinogenic amino acids, 5-hydroxytryptophan residue was determined by COSY correlations between Htrp-NH (δ_H_ 8.22)/Hα (δ_H_ 4.33)/H_2_β (δ_H_ 2.95); H-1 (δ_H_ 10.52)/H-2 (δ_H_ 7.06); H-6 (δ_H_ 6.54)/H-7 (δ_H_ 7.05) and HMBC correlations from Htrp-Hα to Htrp-CO (δ_C_ 171.6); H_2_β to C-3a (δ_C_ 127.9), C-2 (δ_C_ 124.3), C-3 (δ_C_ 108.3); H-1 to C-3; H-2 to C-3a, C-7a (δ_C_ 130.6); H-4 (δ_H_ 6.86) to C-3a, C-5 (δ_C_ 150.4), C-7a; H-6 to C-5; H-7 to C-5 while the other non-proteinogenic amino acid, the *p*-hydroxyphenylglycine, residue was assigned by COSY correlations between Hpg-NH (δ_H_ 8.70)/Hα (δ_H_ 5.23); H-2′ (δ_H_ 6.88)/H-3′ (δ_H_ 6.60); and HMBC correlations from Hα to Hpg-CO (δ_C_ 170.3), C-1′ (δ_C_ 127.9), C-2′ (δ_C_ 129.4); H-2′ to C-4′ (δ_C_ 156.6); H-3′ to C-1′, C-4′ (Figure 2).

In the case of epoxydodecanoic acid moiety, a spin system from H-2 to H_3_-12 was determined based on the serial COSY correlations between those proton signals. Methylene structure was confirmed by the COSY and TOCSY correlations. The MS data and low chemical shift values compared to oxygenated methine group of H-2 (δ_H_ 3.42) and H-3 (δ_H_ 2.81) suggested the existence of a 2,3-epoxide ring. Additionally, the HMBC correlations from H-2 to Epd-CO (δ_C_ 167.8), and the ROESY correlations between H-2/Thr-NH (δ_H_ 7.69) identified the existence of a 2,3-epoxide ring (Figure 2).

The ROESY correlations among the amino acid residues, Thr-Hα (δ_H_ 4.54)/Phe-NH (δ_H_ 8.64); Phe-H-2′ (δ_H_ 7.16)/Glu-Hα (δ_H_ 4.28); Glu-NH (δ_H_ 8.22)/Htrp-Hα; Htrp-Hα/Hpg-NH; and Hpg-Hα/Gly-NH (δ_H_ 8.22) revealed the sequence of amino acids. The downfield resonance of Thr-Hβ (δ_H_ 5.37) with the HMBC correlations from Thr-Hγ (δ_H_ 1.10) to Gly-CO (δ_C_ 168.5) suggested that Thr-Gly were linked by an ester bond. The threonine residue also connected with the 2,3-expoxydodecanoyl moiety assigned to the HMBC correlations from Thr-NH to Epd-CO (Figure 2). Thus, the planar structure of **2** was determined as a cyclic structure with the sequence Thr-Phe-Glu-Htrp-Hpg-Gly, and the peptide moiety connected with the epoxy fatty acid chain.

Cystargamide D (**3**) was isolated as a brownish oil. The UV spectrum and ^1^H NMR chemical shifts of cystargamide D were similar with cystargamides B and C (Table 1). In addition, cystargamide D (**3**) had a similar molecular formula with **1**-**2**, C_47_H_55_N_7_O_13_, based on its HRESI mass spectrometric data and ^1^H and ^13^C NMR data. Accordingly, cystargamide D was expected to be a derivative of **1–2**. Further investigation of the 1D and 2D NMR spectra revealed that cystargamide D is a derivative of **1–2**. The ^1^H and HSQC NMR spectral data indicated that **3** had 8 aliphatic methylene protons.

### Determination of the Absolute Configuration of Cystargamides

The absolute configurations of the amino acids in cystargamides were determined using advanced Marfey’s method (Harada et al., 1996). After oxidation and acid hydrolysis of **1**, the acid hydrolysate of **1** was derivatized with 1-fluoro-2,4-dinitrophenyl-5-L-leucinamide (L-FDLA) and 1-fluoro-2,4-dinitrophenyl-5-D-leucinamide (D-FDLA). The obtained derivatives of **1** were comparatively analyzed by LC/MS with the L-FDLA or D-FDLA derivatives of authentic amino acids. We found that the elution order of derivatized amino acids, Thr, Phe, and Glu, were similar to those of their L-amino acids-L-FDLA derivatives. However, the elution order L-FDLA derivative of the Hpg was opposite to L-standards. To determine the absolute configuration of 5-Htrp, we used the L-Asp as the L-standards of 5-Htrp. The L-Asp-L-FDLA retention time was corresponded to the L-FDLA derivatives of **1** (Supplementary Figures 25, 26 and Supplementary Table 2). The absolute configuration of **2** and **3** were determined by comparison of the circular dichroism (CD) spectra of **1**. The CD spectra of **2** and **3** were nearly identical to that of **1** (Figure 2C). It suggested that cystargamide B, C, and D consist of L-Thr, L-Phe, L-Glu, D-Hpg, and L-Htrp. The relative configuration of vicinal protons in the epoxide ring are assigned as trans by small ^3^*J*_H,H_ coupling constant (1.5 Hz) between H-2 and H-3 of epoxide and the strong ROESY correlations between H-2/Thr-Hα, Thr-Hγ to compared with that between H-3/Thr-Hγ (Figure 2B).

### Genomic Analysis of *Streptomyces* sp. JMS132

**FIGURE 3:**
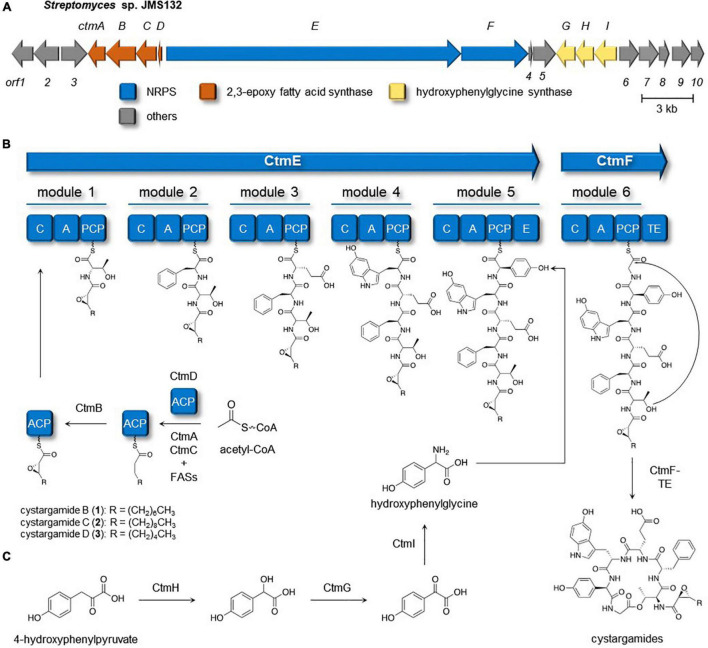
Cystargamides biosynthetic pathway in *Streptomyces* sp. JMS132. **(A)** Biosynthetic gene cluster responsible for production of the cystargamides. **(B)** Putative biosynthetic pathway for the cystargamides. **(C)** Proposed pathway of biosynthesis of hydroxyphenylglycine unit.

Full genome sequencing data of *Streptomyces* sp. JMS132, a cystargamides-producing strain, was acquired to study their biosynthetic pathway. Initial analysis of the genomic data with antiSMASH 6.0 software identified the presence of 23 putative biosynthetic gene clusters (BGCs) including isorenieratene, ectoine, albaflavenone, geosmin (100% similarities), hormaomycins (95% similarity), WS9326s, and hopene (92% similarities) clusters (Table 2; Blin et al., 2021). Further investigations revealed cystargamides-producing cluster (*ctm* cluster) (Figure 3A), with two genes (*ctmE* and *ctmF*) encoding modular nonribosomal peptides synthases (NRPSs), four genes (*ctmA*, *ctmB*, *ctmC*, and *ctmE*) speculated to be responsible for biosynthesizing of trans-2,3-epoxy fatty acids, and three genes (*ctmG*, *ctmH*, and *ctmI*) predicted to produce 4-hydroxyphenylglycine (Hpg) of the cystargamides. In addition, results of adenylation (A) domain analysis of the six modules in the NRPS genes, which indicated Thr-Phe-Glu-Htrp-Hpg-Gly hexapeptide core structures of the cystargamides, and the presence of an additional epimerase (E) domain at downstream of the module for D-Hpg, strongly supported that the cluster (*ctm* cluster) is supposed to produce cystargamides. Further gene deletion studies are necessary to verify this cluster as the origin of the cystargamide natural products.

**TABLE 2:** Putative functions of the open reading frames in the biosynthetic gene cluster of cystargamides in *Streptomyces* sp. JSM132.

Gene product	Size (AA)	Best match		
		Putative function [Organism]	Accession number	Identity (%)/Similarity (%)
Orf1	424	VWA domain-containing protein [*Streptomyces flaveolus*]	WP_189232053.1	96/97
Orf2	499	IucA/IucC family siderophore biosynthesis protein [*Streptomyces* sp. SID5910]	WP_237518971.1	85/88
Orf3	512	IucA/IucC family siderophore biosynthesis protein [*Streptomyces solaniscabiei*]	WP_228754323.1	90/91
CtmA	343	Ketoacyl-ACP synthase III [*Streptomyces* sp. TSRI0107]	WP_073939330.1	90/93
CtmB	591	Acyl-CoA dehydrogenase family protein [*Streptomyces* sp. TSRI0107]	WP_079186740.1	85/91
CtmC	405	Beta-ketoacyl-[acyl-carrier-protein] synthase family protein [*Streptomyces* sp. TSRI0107]	WP_073939328.1	87/93
CtmD	81	Acyl carrier protein [*Streptomyces* sp. TSRI0107]	WP_073939327.1	78/86
CtmE	5748	NRPS (C-A-PCP-C-A-PCP-C-A-PCP-C-A-PCP-C-A-PCP-E)		
CtmF	1310	NRPS (C-A-PCP-TE)		
Orf4	75	MbtH family protein [*Streptomyces* sp. TSRI0107]	WP_073939324.1	92/96
Orf5	449	Hypothetical protein [*Streptomyces* sp. TSRI0107]	WP_079186710.1	85/88
CtmG	373	Alpha-hydroxy-acid oxidizing protein [*Streptomyces* sp. CAI-121]	NUV70431.1	86/91
CtmH	363	MULTISPECIES: 4-hydroxyphenylpyruvate dioxygenase [unclassified *Streptomyces*]	WP_175467738.1	87/91
CtmI	442	PLP-dependent aminotransferase family protein [*Streptomyces* sp. TSRI0107]	WP_073939321.1	86/90
Orf6	386	c-type cytochrome biogenesis protein CcsB [*Streptomyces* sp. TSRI0107]	WP_073939320.1	88/91
Orf7	383	LLM class flavin-dependent oxidoreductase [*Streptomyces* sp. SID8352]	WP_161227768.1	91//96
Orf8	202	MULTISPECIES: NAD(P)H-dependent oxidoreductase [unclassified *Streptomyces*]	WP_161227768.1	85/89
Orf9	367	Hemin transport system permease protein HmuU [*Streptomyces* sp. 111WW2]	PSK58834.1	91/94
Orf10	257	ATP-binding cassette domain-containing protein [*Streptomyces* sp. AC558_RSS880]	WP_217133345.1	89/93

### Cystargamides Biosynthetic Pathway in *Streptomyces* sp. JMS132

The biosynthetic procedure of trans-2,3-epoxy fatty acids, one of the characteristic moieties in the cystargamides, was proposed based on a comparative analysis of previous studies on the biosynthesis of calcium-dependent antibiotics (CDAs) (Hojati et al., 2002), which also contain trans-2,3-epoxyacyl moiety in their structure (Figure 3B). In the middle of CDAs biosynthetic pathway, FabF3, FabH4, β-ketoacyl-ACP synthases (KAS)-II, and KAS-III, respectively, were reported to be responsible for the production of a hexanoyl-ACP together with several fatty acid synthases (FASs) from primary metabolism. Then, two epoxidases in the CDAs-producing cluster, HxcO and HcmO, are predicted to play a role in desaturation and epoxidation of the acyl-ACP to generate trans-2,3-epoxyhexanoic acid. During a detailed investigation on *ctm* cluster, *ctmC*, *ctmA*, and *ctmB* were identified to be homologous with *fabF3*, *fabH4*, and *hxcO*, respectively. However, no genes homologous with *hcmO* were discovered from the cluster, so it could be proposed that only CtmB (HxcO-like protein) is utilized for the production of trans-2,3-epoxy fatty acid during the biosynthesis of the cystargamides.

Biosynthesis of 4-hydroxyphenylglycine, a non-proteinogenic amino acid building block in cystargamides, was also predicted based on the detailed genomic investigation on the *ctm* cluster and previous research studies on the biosynthesis of hydroxyphenylglycine in CDAs and vancomycins (Choroba et al., 2000; Hubbard et al., 2000; Hojati et al., 2002). Initially, 4-hydroxyphenylpyruvate originating from L-tyrosine or prephenate is converted into 4-hydroxymandelate by 4-hydroxymandelate synthase (CtmH), and 4-hydroxymandelate oxidase (CtmG) and 4-hydroxyphenylglycine aminotransferase (CtmI) in the *ctm* cluster subsequently oxidize and transaminase the intermediates, respectively, yielding L-4-hydroxyphenylglycine residue, which is epimerized after being incorporated in the NRPS procedure (Figure 3C).

### Antioxidant Activity of Cystargamides

**FIGURE 4:**
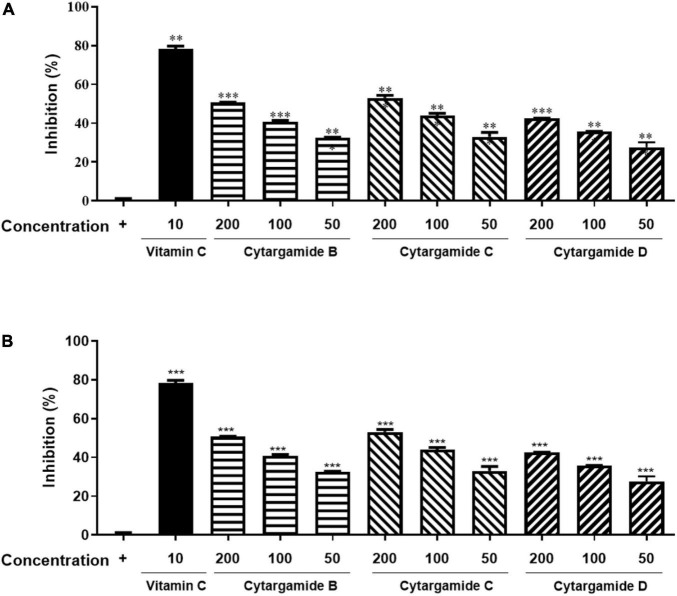
Antioxidant activities of cystargamides. **(A)** Cystargamides and vitamin C were mixed with DPPH radical substrate (0.3 mM) for 30 min. The DPPH radical scavenging effect was measured by the absorbance at 490 nm using an ELISA microplate reader. **(B)** Cystargamides and vitamin C were mixed with ABTS cation radical substrate (7.4 mM) for 30 min. The ABTS radical scavenging effect was measured by the absorbance at 750 nm using an ELISA microplate reader. DPPH radicals were decreased in a dose-dependent manner by cystargamide treatment. Data were expressed as mean ± SEM. **p < 0.05 and ***p < 0.001 compared with the control group.

The antioxidant activity of cystargamides was evaluated by DPPH, and ABTS radical scavenging effects. Vitamin C was used as a positive antioxidant control drug. First, cystargamides B, C, and D (**1–3**) showed significant scavenging activity on DPPH free radicals in a dose-dependent manner. Cystargamide C (**2**) at a concentration of 200 μg/mL decreased DPPH free radicals by approximately 53% (Figure 4A). The cystargamides also showed strong scavenging activity on ABTS cation radicals comparable to the vitamin C positive control (Figure 4B). Cystargamide D (**3**) at a concentration of 200 μg/mL decreased ABTS free radicals by 100%. Our study results indicate that cystargamides B, C, and D have significant antioxidant activity.

## Conclusion

LC/MS based chemical investigation results of tidal mudflat-derived bacteria showed two new lipodepsipeptides, cystargamides C and D (**2–3**). Based on 1/2D NMR spectroscopy, Marfey’s method, and CD analysis, the two new compounds were determined to have six amino acids (L-Thr, L-Phe, L-Glu, L-Htrp, D-Hpg, and Gly) and an epoxy fatty acid moiety. Cystargamides C and D had a close similarity to the previously discovered cystargamides A and B (Gill et al., 2014; Kitani et al., 2018), but the length of the epoxy acid lipid chain in **2** and **3** was different from that in cystargamides A and B. Full genomic analysis of the strain identified biosynthetic gene clusters of cystargamide and revealed biosynthetic pathway integrated nonribosomal peptide synthase and trans 2,3-epoxy fatty acid synthesis. Studies on the biological activities of cystargamides are limited, this is the first report on the evaluation of antioxidant effects of **1–3**. Our discovery of the two new lipodepsipeptides from a tidal mudflat-derived bacteria suggested that microorganisms inhabiting diverse marine environments are potent sources of marine drugs.

## Data Availability Statement

The original contributions presented in the study are included in the article/Supplementary Material, further inquiries can be directed to the corresponding author.

## Author Contributions

JS cultured and isolated the compounds. Y-HS conducted genomic analysis of the strain. SJ evaluated the biological activities. All authors designed the experiments and approved the submitted version.

## Conflict of Interest

The authors declare that the research was conducted in the absence of any commercial or financial relationships that could be construed as a potential conflict of interest.

## Publisher’s Note

All claims expressed in this article are solely those of the authors and do not necessarily represent those of their affiliated organizations, or those of the publisher, the editors and the reviewers. Any product that may be evaluated in this article, or claim that may be made by its manufacturer, is not guaranteed or endorsed by the publisher.
