# Supplementary material for: Cystargamides C and D, New Cyclic Lipopeptides From a Tidal Mudflat-Derived Streptomyces sp. JMS132
Source: Front Microbiol. 2022 May 13;13:904954. doi: 10.3389/fmicb.2022.904954 (PMC9136287; doi:10.3389/fmicb.2022.904954)
Supplement: Supplementary file 1 [file Data_Sheet_1.pdf]

Supplementary Material

Cystargamides C and D, new cyclic lipopeptides from a tidal mud flat-derived *Streptomyces* sp. JMS 132

Jeongwon Seo[1], Yern-Hyerk Shin[2], Se Jin Jo[1], Young Eun Du[3], Soohyun Um[4], Young Ran Kim[1], and Kyuho Moon[1,*]

[1]College of Pharmacy, Research Institute of Pharmaceutical Sciences, Chonnam National University, Gwangju 61186, Republic of Korea

[2]Department of Biological Chemistry and Molecular Pharmacology, Harvard Medical School and Blavatnik Institute, Boston, MA 02115, USA

[3]Natural Products Research Institute, College of Pharmacy, Seoul National University, Seoul 08826, Republic of Korea

[4]College of Pharmacy, Yonsei University, Incheon 21983, Republic of Korea

***Correspondence:**
Corresponding Author
khmoon@jnu.ac.kr

Table of Contents

Figure S1. ^{1}H NMR spectrum of cystargamide B (**1**) at 900 MHz in DMSO-d_6.

Figure S2. ^{13}C NMR spectrum of cystargamide B (**1**) at 225 MHz in DMSO-d_6.

Figure S3. HSQC NMR spectrum of cystargamide B (**1**) at 900 MHz in DMSO-d_6.

Figure S4. TOCSY NMR spectrum of cystargamide B (**1**) at 900 MHz in DMSO-d_6.

Figure S5. COSY NMR spectrum of cystargamide B (**1**) at 900 MHz in DMSO-d_6.

Figure S6. HMBC NMR spectrum of cystargamide B (**1**) at 900 MHz in DMSO-d_6.

Figure S7. ROESY NMR spectrum of cystargamide B (**1**) at 900 MHz in DMSO-d_6.

Table S1. NMR spectral data of cystargamide B (**1**) in DMSO-d_6

Figure S8. ^{1}H NMR spectrum cystargamide C (**2**) at 900 MHz in DMSO-d_6.

Figure S9. ^{13}C NMR spectrum cystargamide C (**2**) at 225 MHz in DMSO-d_6.

Figure S10. HSQC NMR spectrum cystargamide C (**2**) at 900 MHz in DMSO-d_6.

Figure S11. TOCSY NMR spectrum cystargamide C (**2**) at 900 MHz in DMSO-d_6.

Figure S12. COSY NMR spectrum cystargamide C (**2**) at 900 MHz in DMSO-d_6.

Figure S13. HMBC NMR spectrum cystargamide C (**2**) at 900 MHz in DMSO-d_6.

Figure S14. ROESY NMR spectrum cystargamide C (**2**) at 900 MHz in DMSO-d_6.

Figure S15. ^{1}H NMR spectrum cystargamide D (**3**) at 700 MHz in DMSO-d_6.

Figure S16. ^{13}C NMR spectrum cystargamide D (**3**) at 175 MHz in DMSO-d_6.

Figure S17. HSQC NMR spectrum cystargamide D (**3**) at 700 MHz in DMSO-d_6.

Figure S18. TOCSY NMR spectrum cystargamide D (**3**) at 700 MHz in DMSO-d_6.

Figure S19. COSY NMR spectrum cystargamide D (**3**) at 700 MHz in DMSO-d_6.

Figure S20. HMBC NMR spectrum cystargamide D (**3**) at 700 MHz in DMSO-d_6.

Figure S21. ROESY NMR spectrum cystargamide D (**3**) at 700 MHz in DMSO-d_6.

Figure S22. HR-ESI-MS data of cystargamide B (**1**).

Figure S23. HR-ESI-MS data of cystargamide C (**2**).

Figure S24. HR-ESI-MS data of cystargamide D (**3**).

Figure S25. Extracted-ion chromatograms of L- and D-FDLA derivatives of amino acid standards.

Figure S26. Extracted-ion chromatograms of L- and D-FDLA derivatized hydrolysate of cystargamide B (**1**).

Table S2. LC/MS analysis data of L- and D-FDLA derivatives of amino acids.

Table S3. antiSMASH output table of *Streptomyces* sp. JMS132.

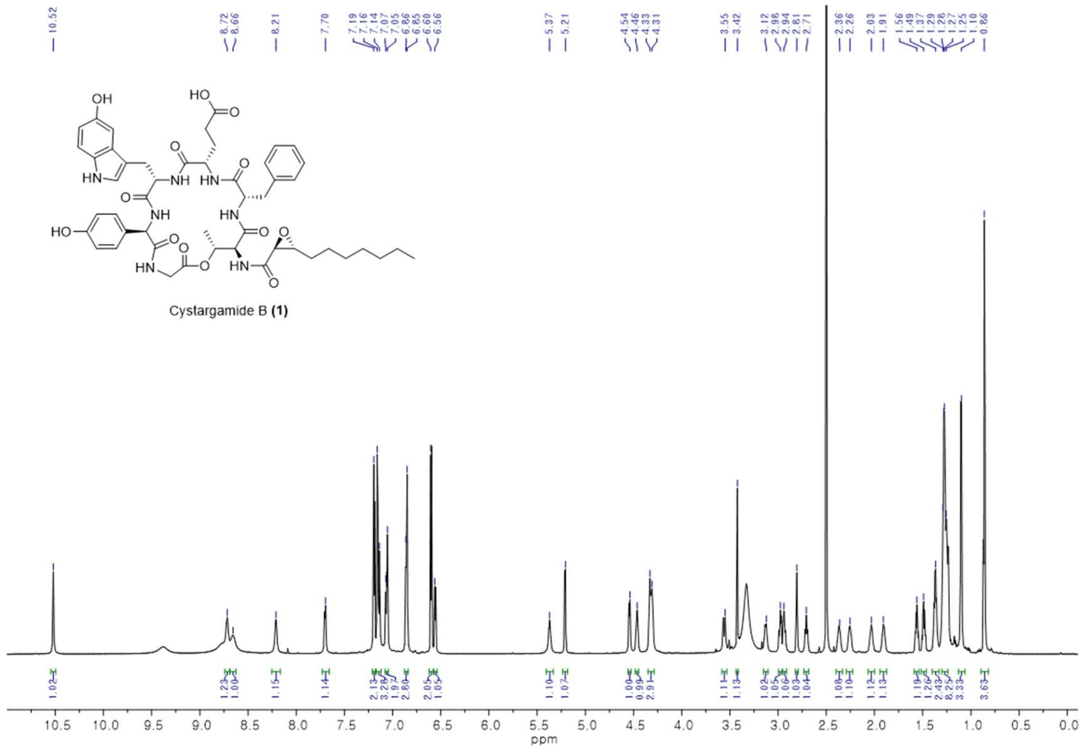

Figure S1. ^{1}H NMR spectrum of cystargamide B (**1**) at 900 MHz in DMSO-d_6.

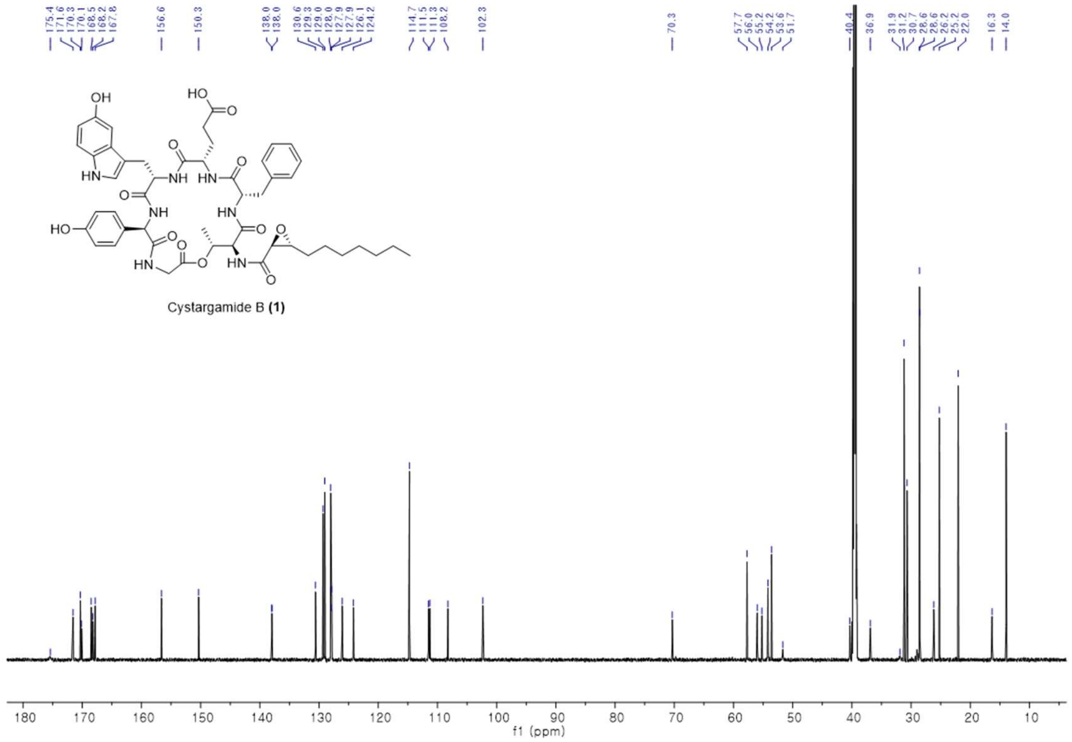

Figure S2. ^{13}C NMR spectrum of cystargamide B (**1**) at 225 MHz in DMSO-d_6.

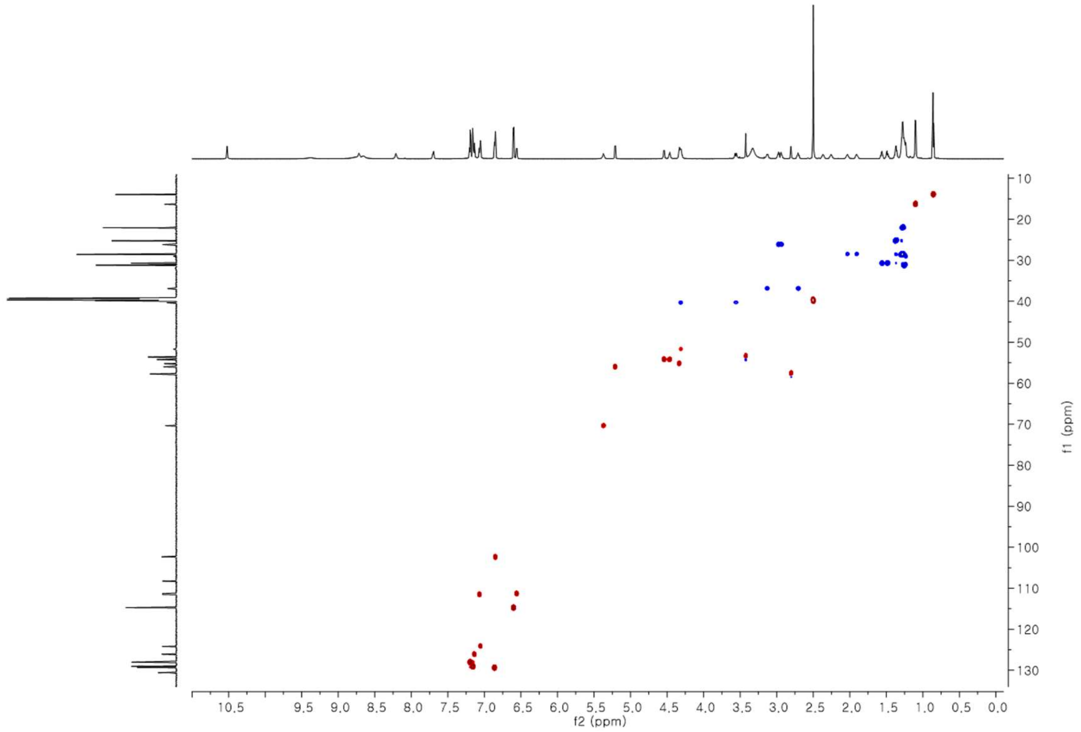

Figure S3. HSQC NMR spectrum of cystargamide B (**1**) at 900 MHz in DMSO-d_6.

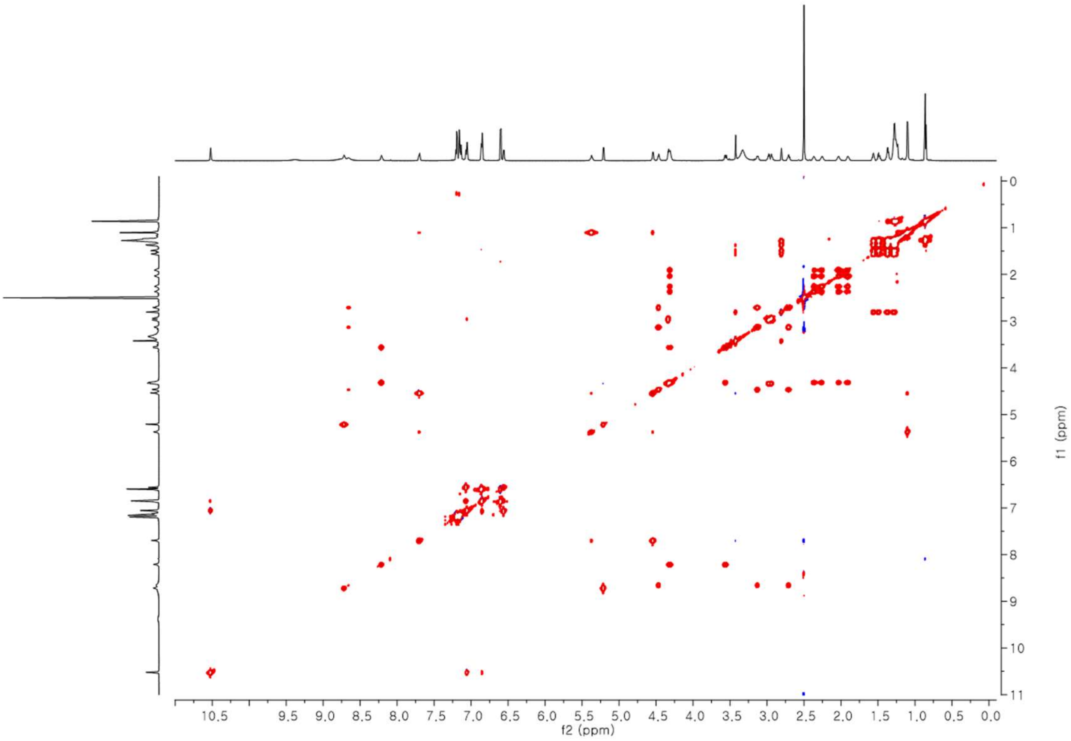

Figure S4. TOCSY NMR spectrum of cystargamide B (**1**) at 900 MHz in DMSO-d_6.

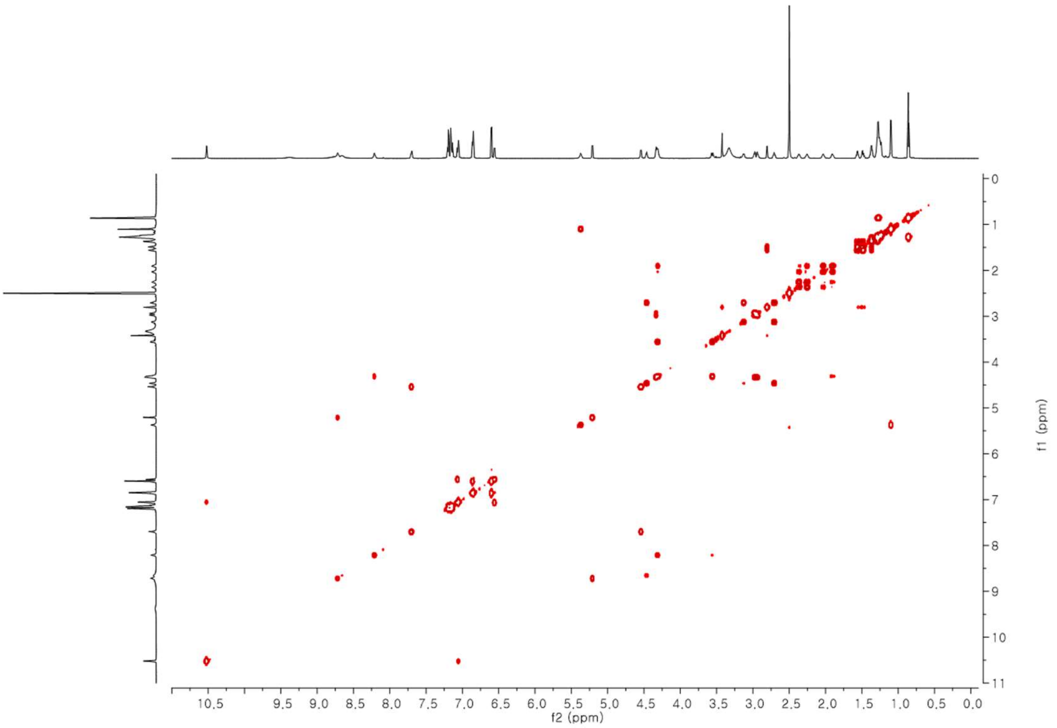

Figure S5. COSY NMR spectrum of cystargamide B (**1**) at 900 MHz in DMSO-d_6.

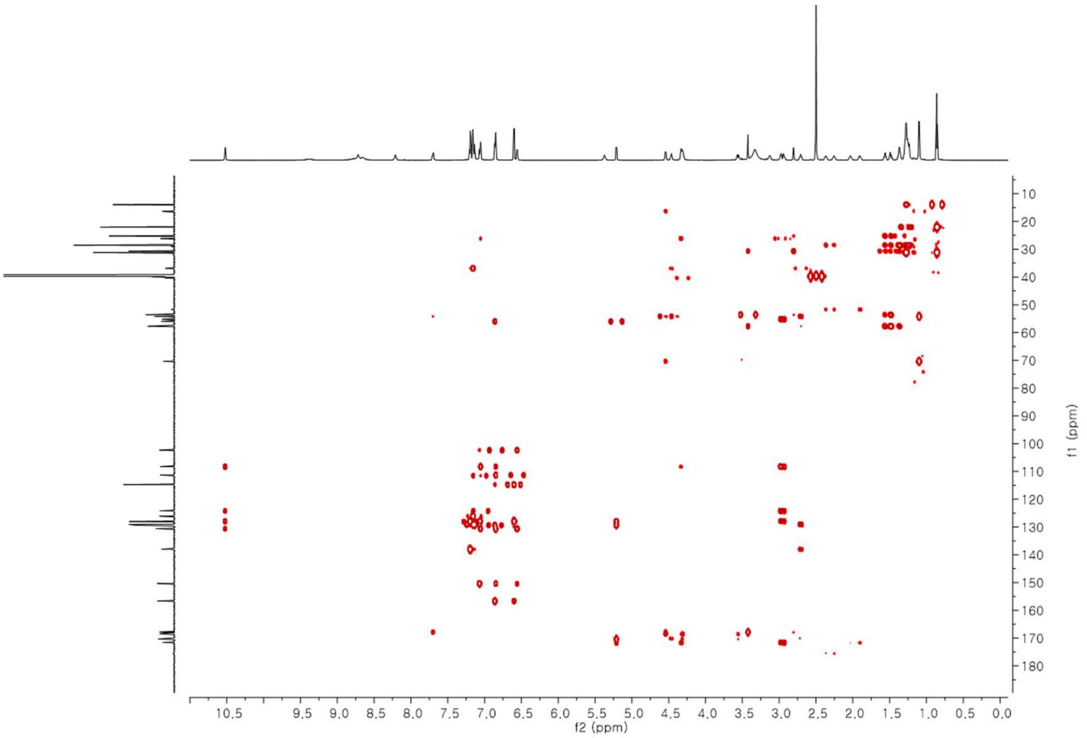

Figure S6. HMBC NMR spectrum of cystargamide B (**1**) at 900 MHz in DMSO-d_6.

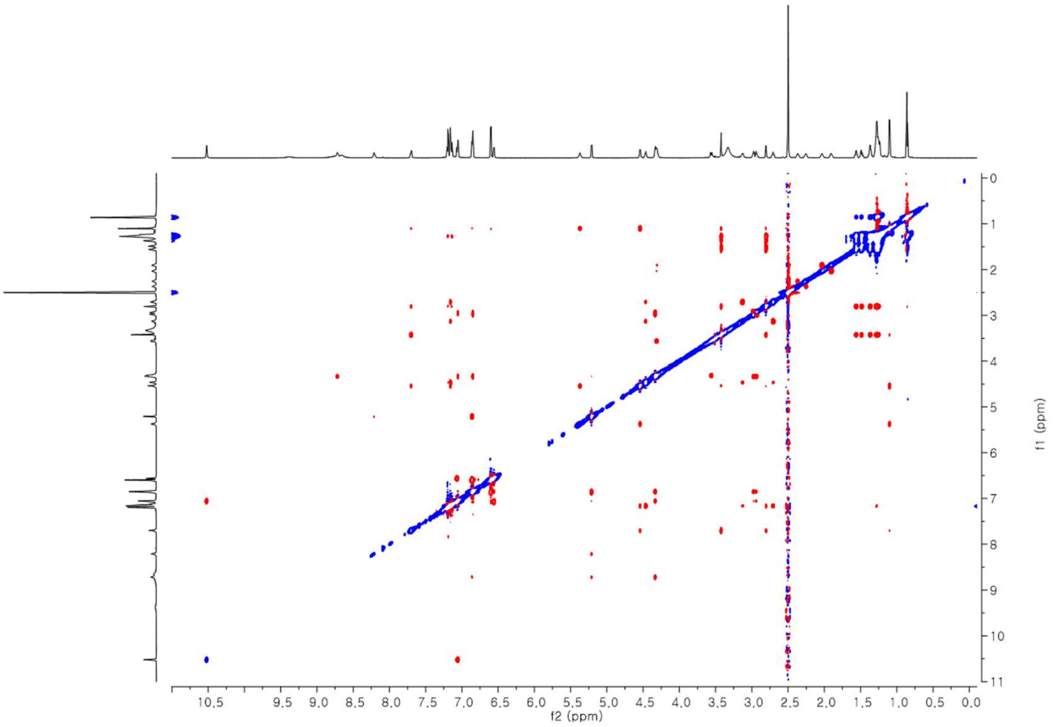

Figure S7. ROESY NMR spectrum of cystargamide B (**1**) at 900 MHz in DMSO-d_6.

Table S1. NMR spectral data of cystargamide B (**1**) in DMSO-d_6[a]

position		δ_C, type	δ_H, mult (J, Hz)
Epd	CO	167.8, C	
	2	53.6, CH	3.42, d (1.5)
	3	57.7, CH	2.81, dt (4.5, 1.5)
	4	30.7, CH_2	1.56, m; 1.49, m
	5[b]	25.2, CH_2	1.37, m; 1.29, m
	6[b]	28.6, CH_2	1.37, m; 1.28, m
	7[b]	28.6, CH_2	1.28, m
	8[b]	31.2, CH_2	1.25, m
	9[b]	22.0, CH_2	1.27, m
	10[b]	14.0, CH_3	0.86, t (7.0)
Thr	CO	168.2, C	
	NH		7.70, d (8.5)
	α	54.2, CH	4.54, d (9.0)
	β	70.3, CH	5.37, m
	γ	16.3, CH_3	1.10, d (6.0)
Phe	CO	170.1, C	
	NH		8.66, m
	α	54.2, CH	4.46, m
	β	36.9, CH_2	3.12, m; 2.71, m
	1'	138.0, C	
	2'/6'	129.0, CH	7.16, m
	3'/5'	128.0, CH	7.19, m
	4'	126.1, CH	7.14, m
Glu	CO	171.6, C	
	NH		8.21, m
	α	51.7, CH	4.31, m
	β	28.6, CH_2	2.03, m; 1.91, m
	γ	31.9, CH_2	2.36, m; 2.26, m
	COOH	175.4, C	
Htrp	CO	171.6, C	

	NH		8.21, m
	α	55.2, CH	4.33, m
	β	26.2, CH_2	2.98, dd (14.0, 8.0); 2.94, dd (14.0, 7.0)
	1		10.52, m
	2	124.2, CH	7.05, m
	3	108.2, C	
	3a	127.9, C	
	4	102.3, CH	6.85, m
	5	150.3, C	
	6	111.3, CH	6.56, m
	7	111.5, CH	7.07, m
	7a	130.6, C	
Hpg	CO	170.3, C	
	NH		8.72, m
	α	56.0, CH	5.21, d (8.0)
	1'	128.0, C	
	2'/6'	129.3, CH	6.86, m
	3'/5'	114.7, CH	6.60, m
	4'	156.6, C	
Gly	CO	168.5, C	
	NH		8.21, m
	α	40.4, CH_2	4.31, m; 3.56, m

[a] ^1H and ^{13}C data were recorded at 900 and 225 MHz, respectively.

[b] Overlapped signals.

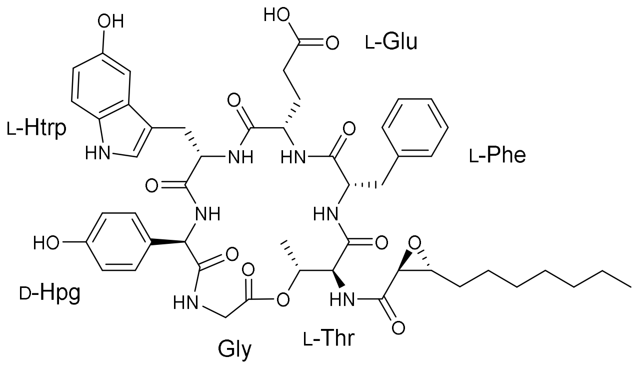

Cystargamide B **(1)**

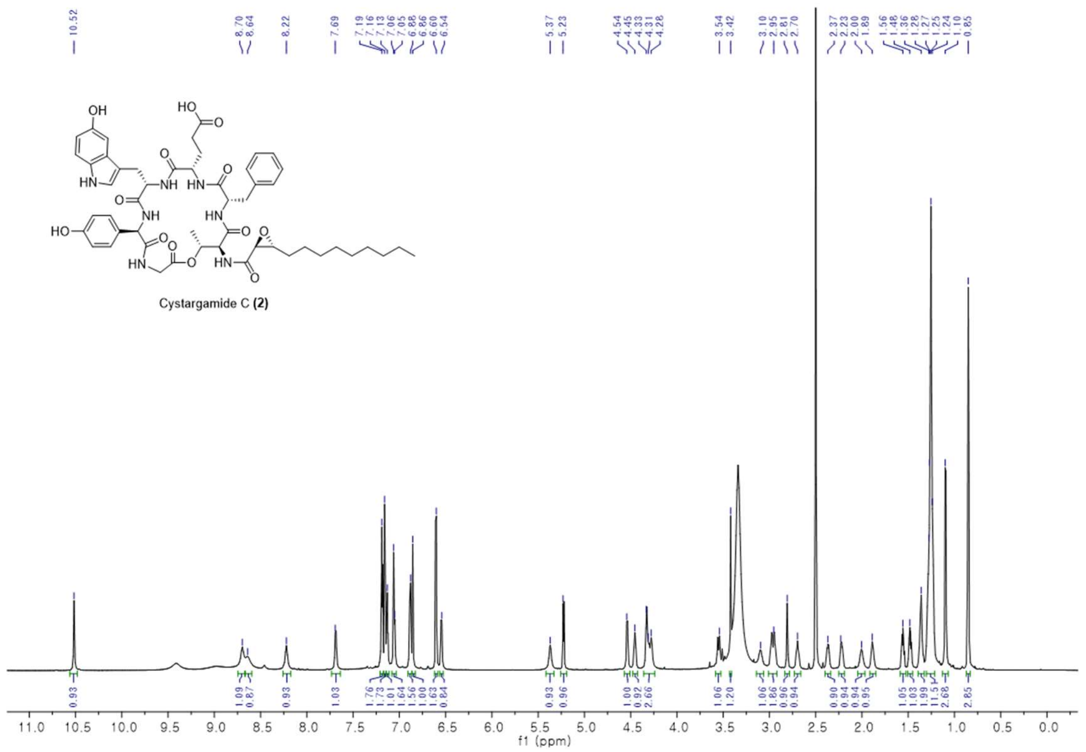

Figure S8. ^{1}H NMR spectrum of cystargamide C (**2**) at 900 MHz in DMSO-d_6.

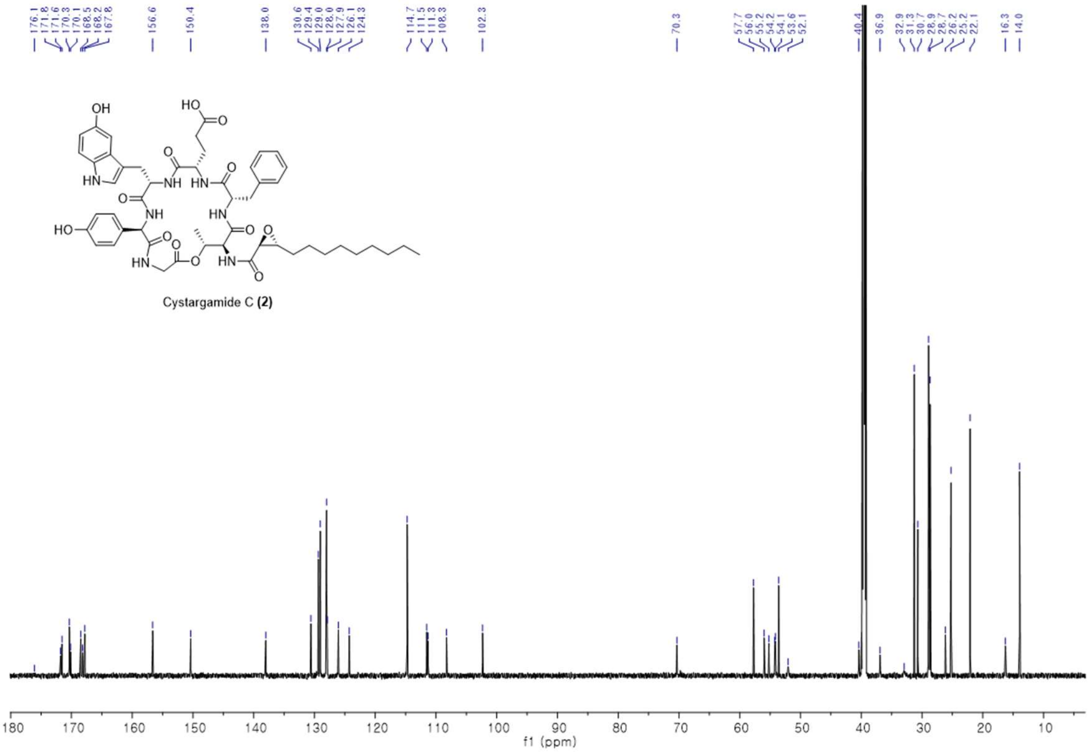

Figure S9. ^{13}C NMR spectrum of cystargamide C (**2**) at 225 MHz in DMSO-d_6.

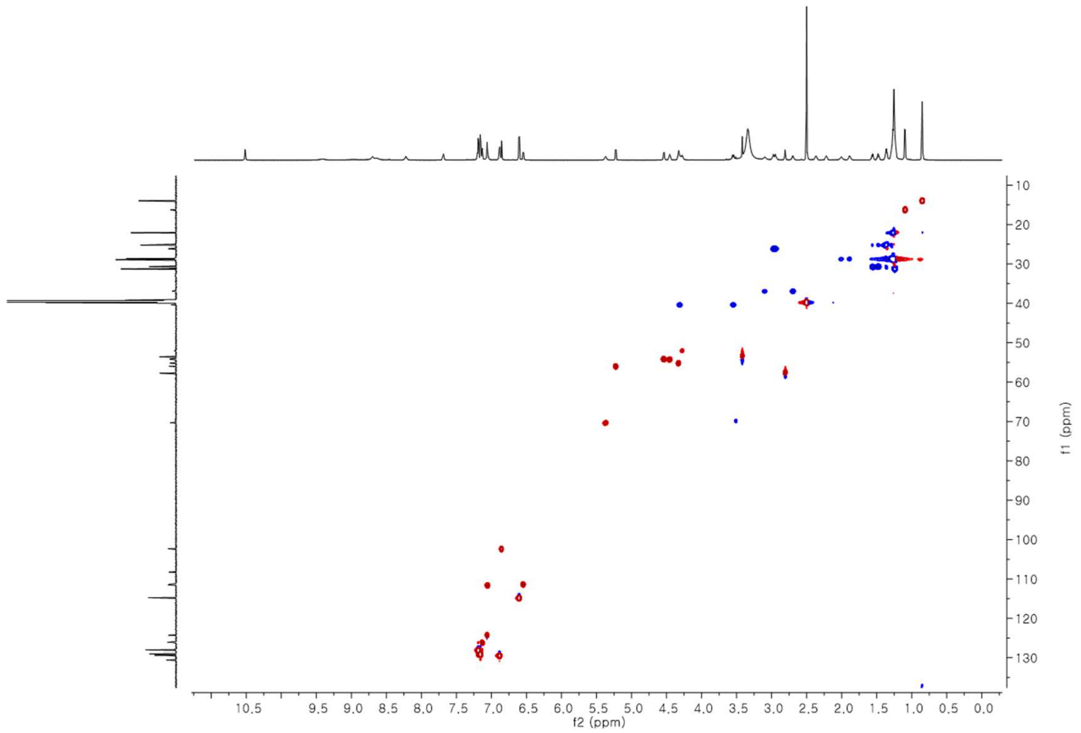

Figure S10. HSQC NMR spectrum of cystargamide C (**2**) at 900 MHz in DMSO-d_6.

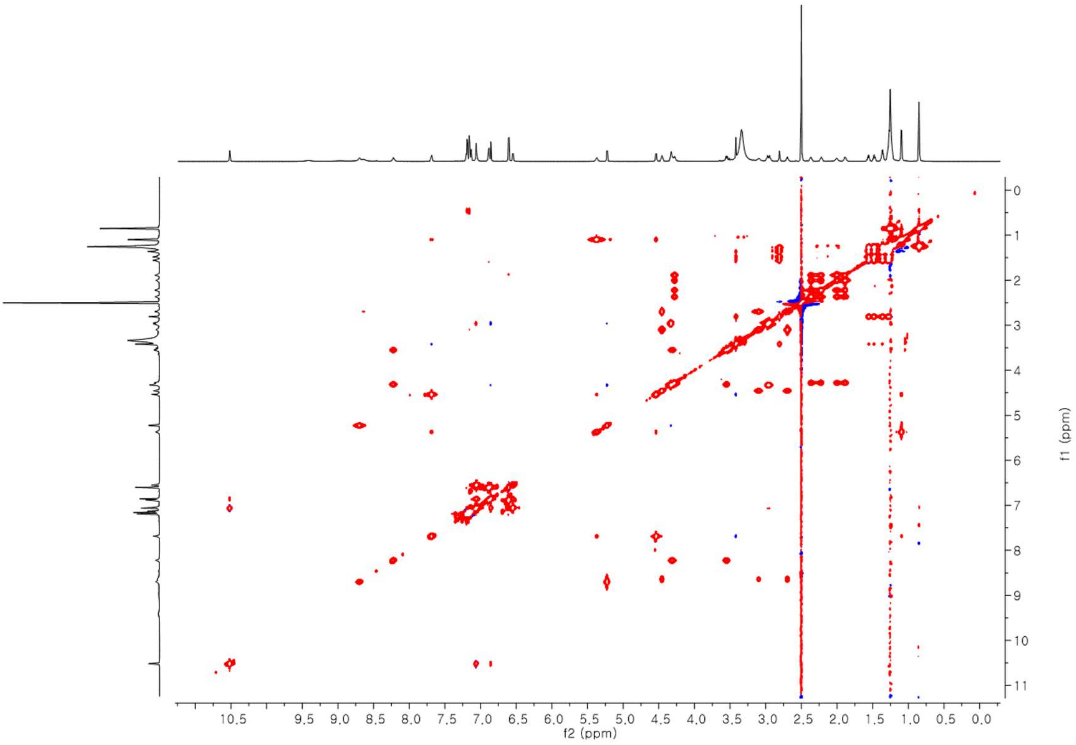

Figure S11. TOCSY NMR spectrum of cystargamide C (**2**) at 900 MHz in DMSO-d_6.

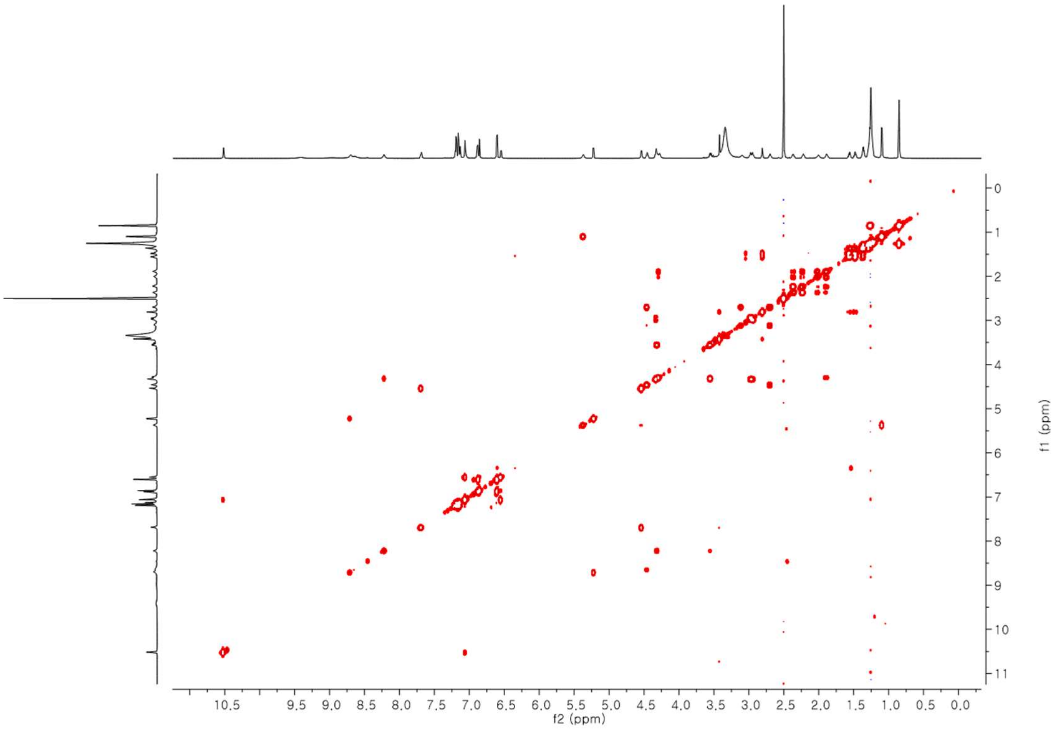

Figure S12. COSY NMR spectrum of cystargamide C (**2**) at 900 MHz in DMSO-d_6.

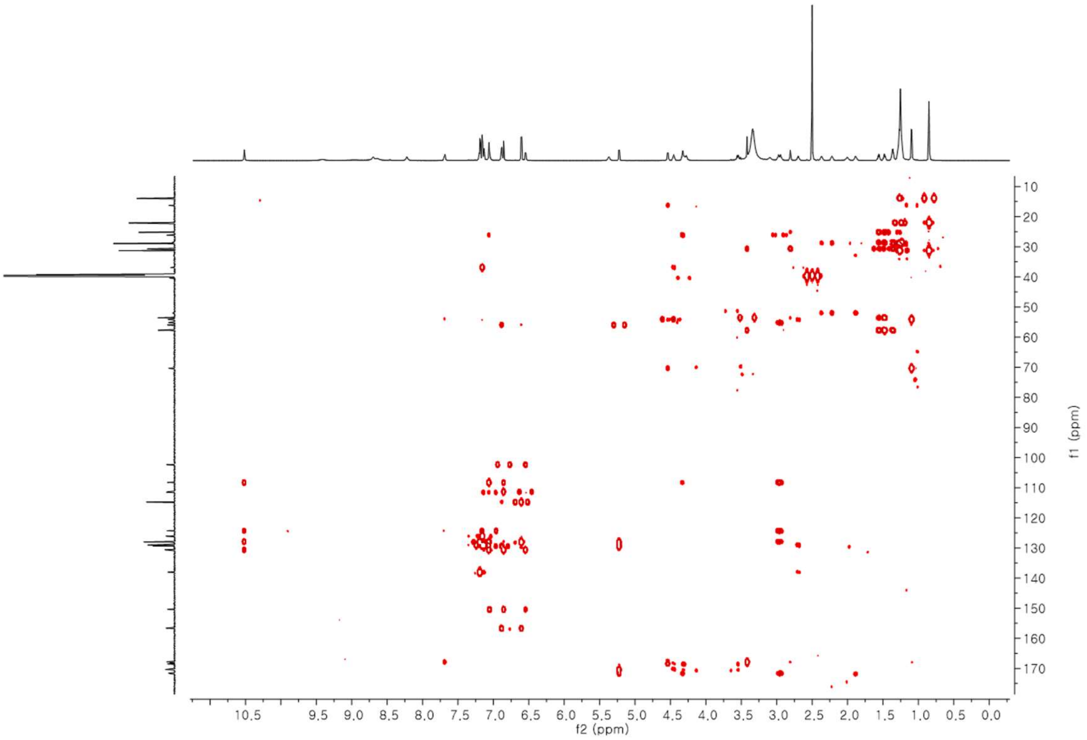

Figure S13. HMBC NMR spectrum of cystargamide C (**2**) at 900 MHz in DMSO-d_6.

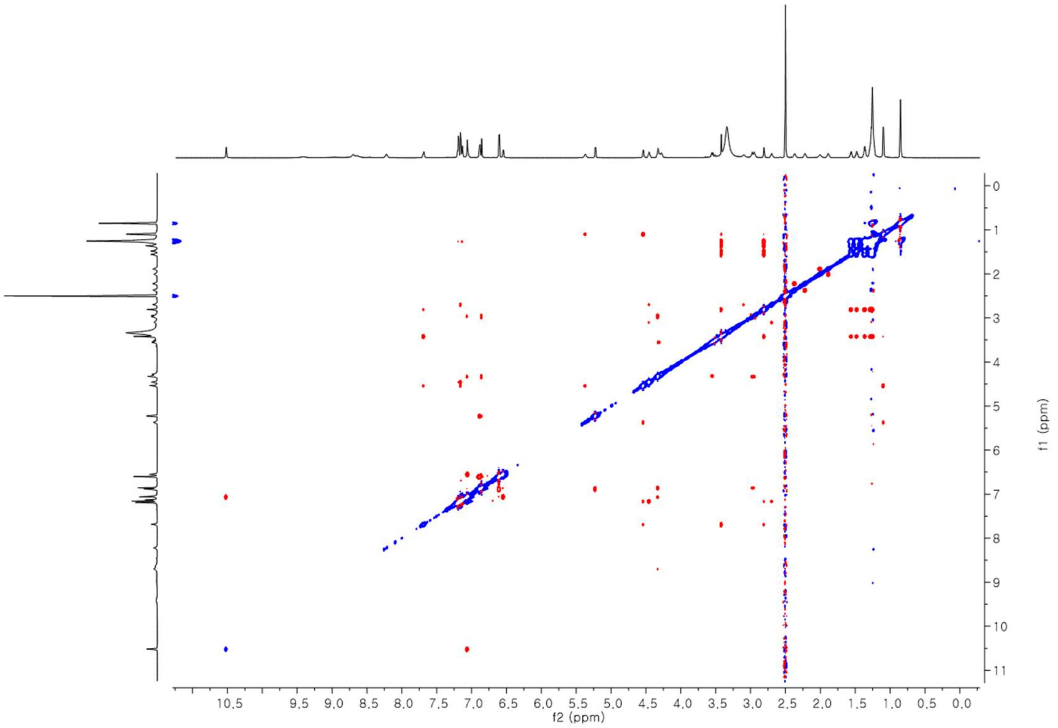

Figure S14. ROESY NMR spectrum of cystargamide C (**2**) at 900 MHz in DMSO-d_6.

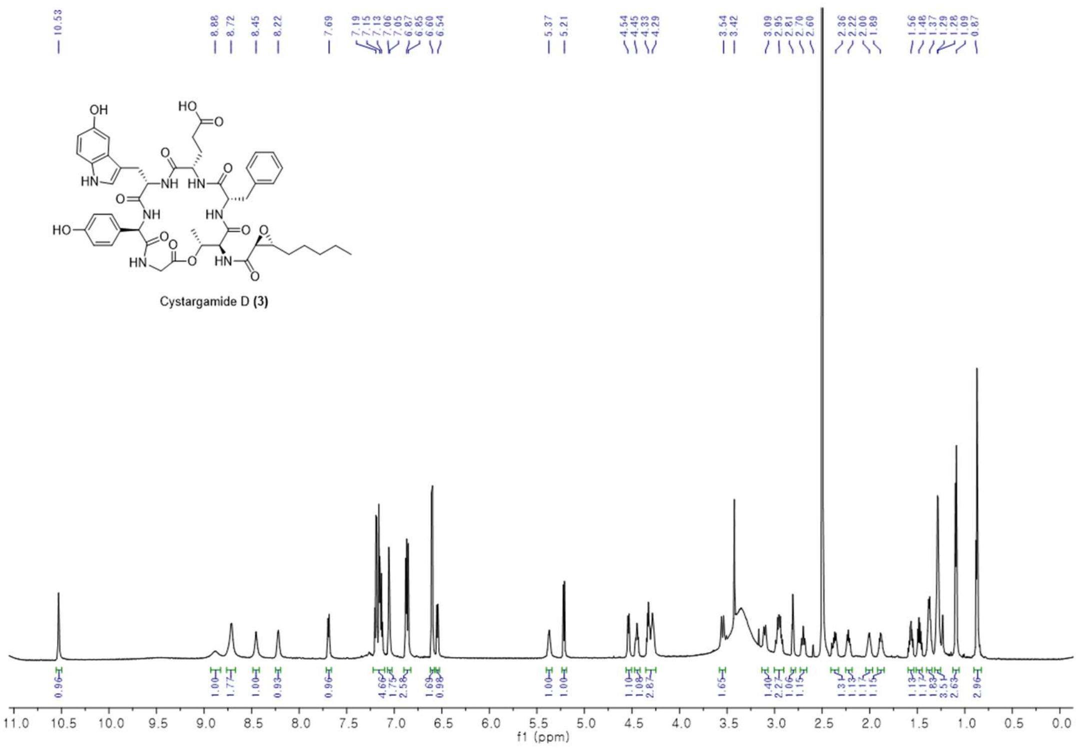

Figure S15. ^{1}H NMR spectrum of cystargamide D (**3**) at 700 MHz in DMSO-d_6.

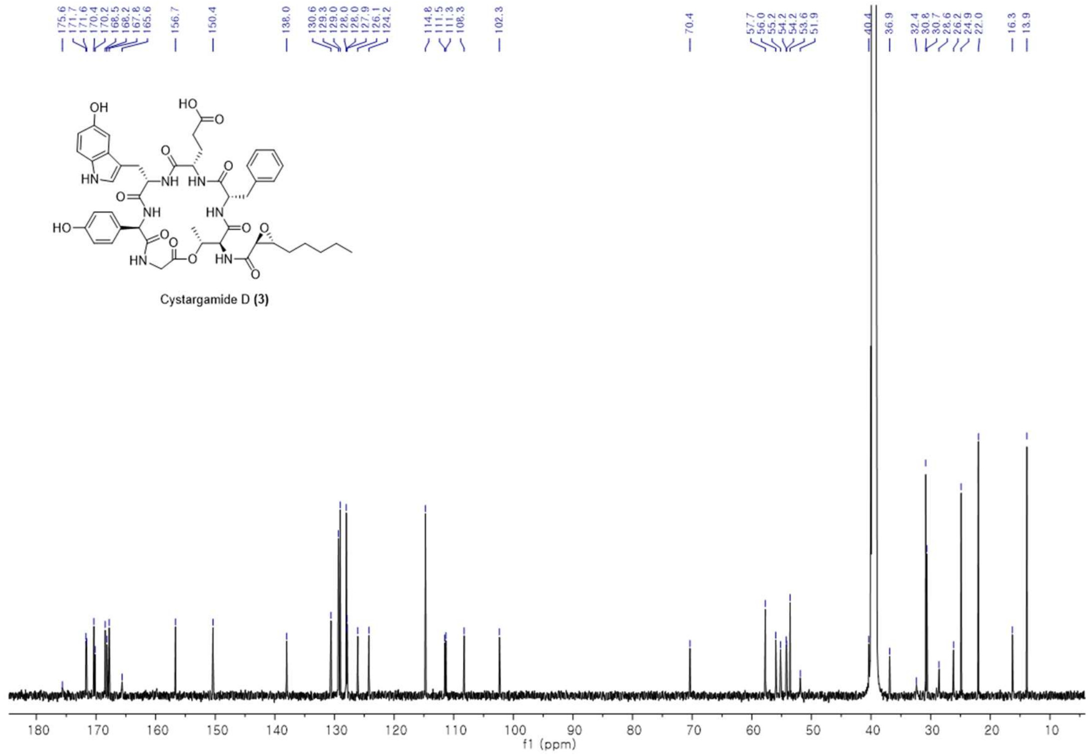

Figure S16. ^{13}C NMR spectrum of cystargamide D (**3**) at 175 MHz in DMSO-d_6.

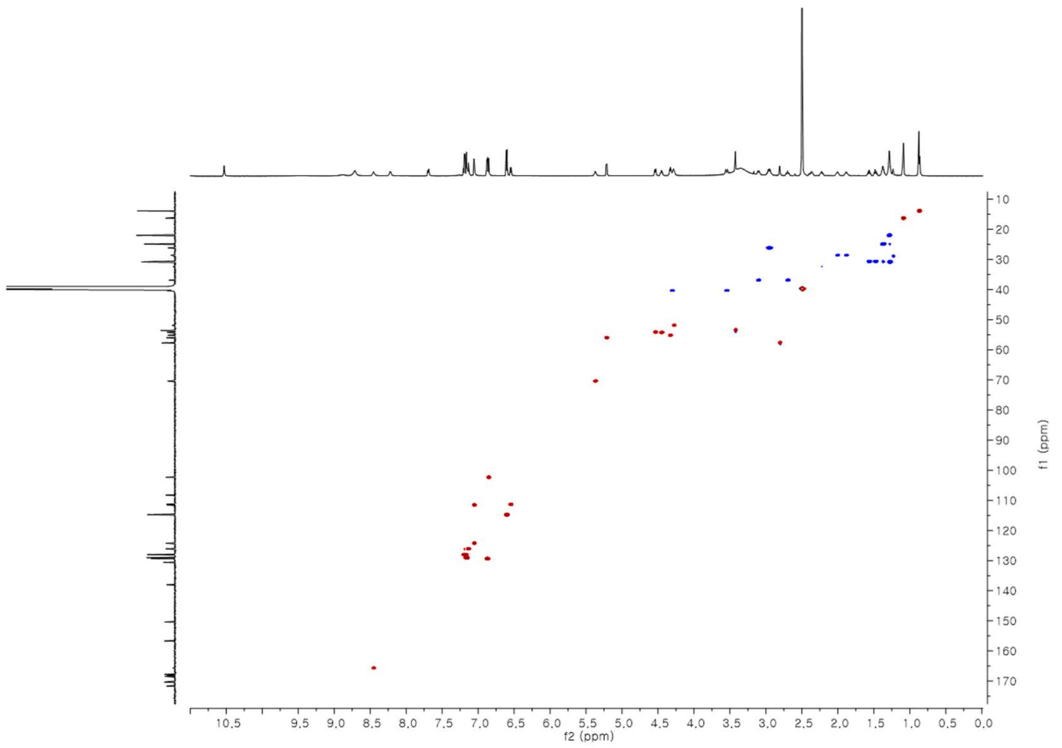

Figure S17. HSQC NMR spectrum of cystargamide D (**3**) at 700 MHz in DMSO-d_6.

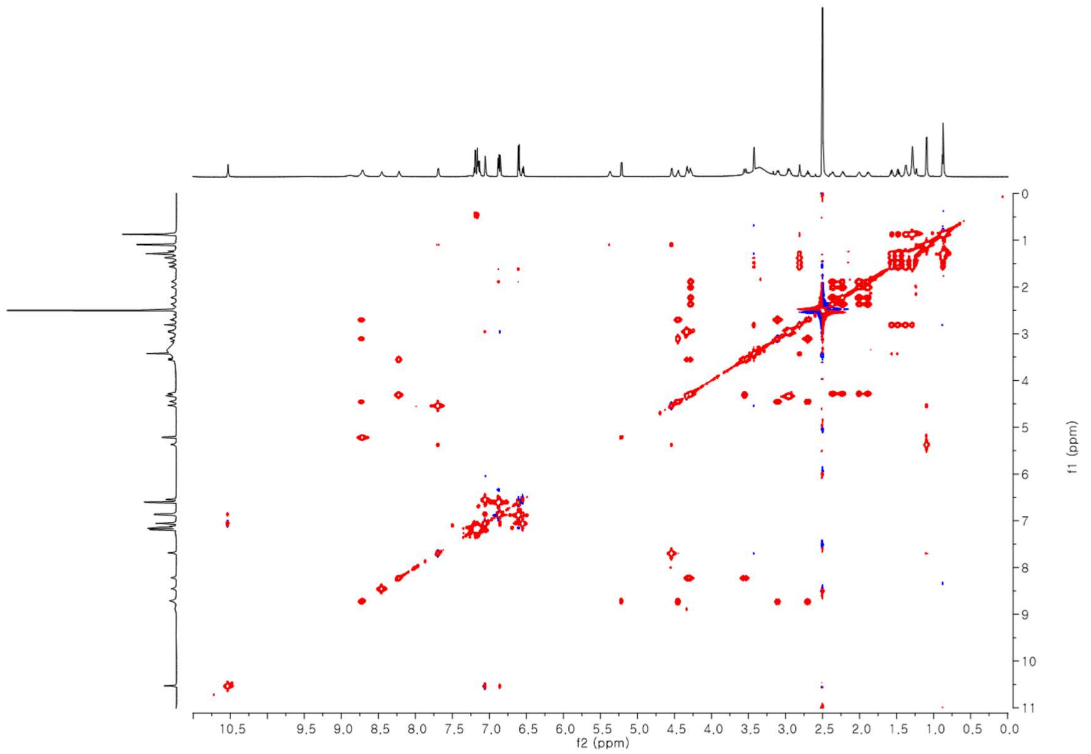

Figure S18. TOCSY NMR spectrum of cystargamide D (**3**) at 700 MHz in DMSO-d_6.

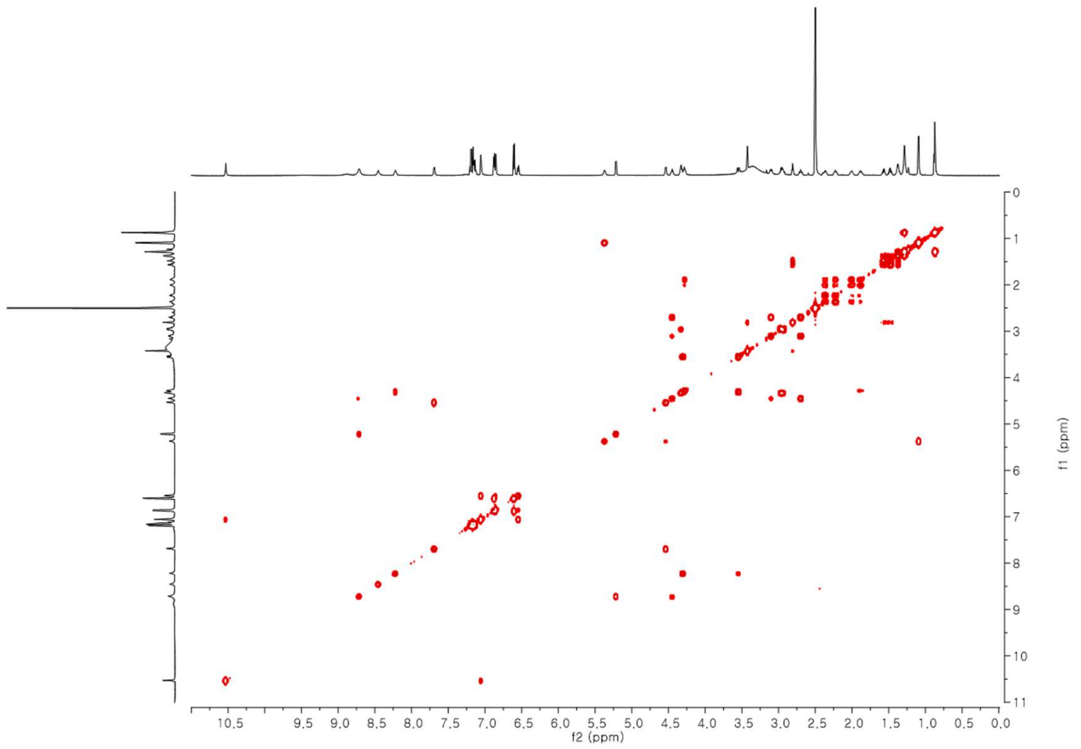

Figure S19. COSY NMR spectrum of cystargamide D (**3**) at 700 MHz in DMSO-d_6.

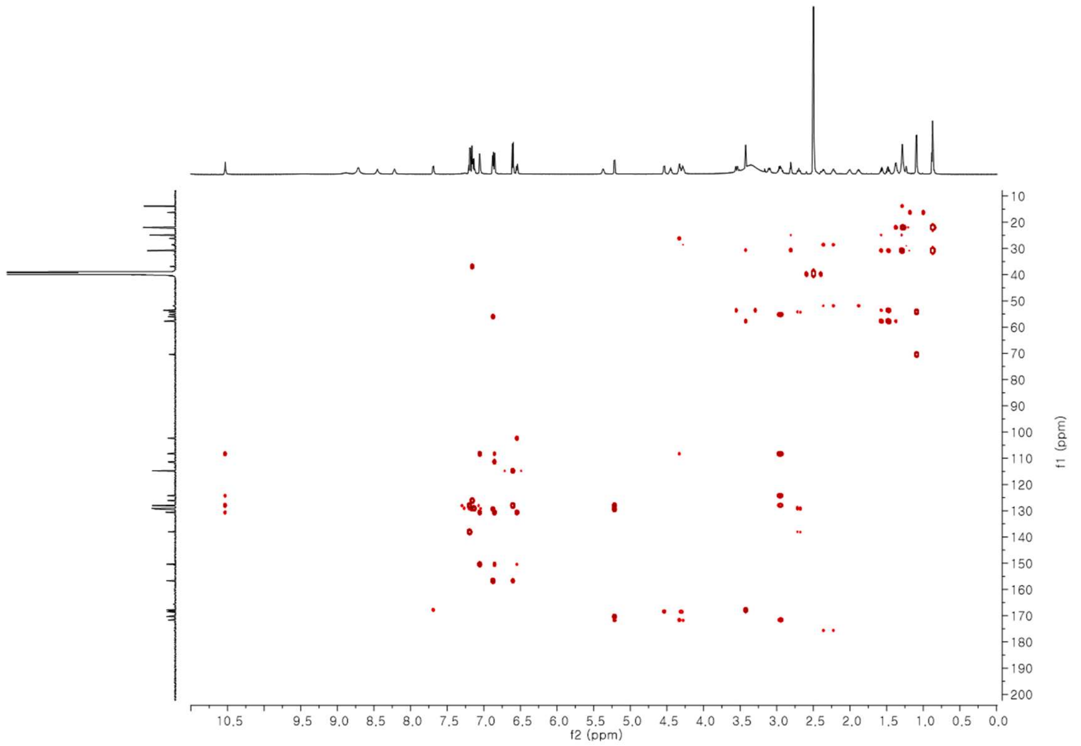

Figure S20. HMBC NMR spectrum of cystargamide D (**3**) at 700 MHz in DMSO-d_6.

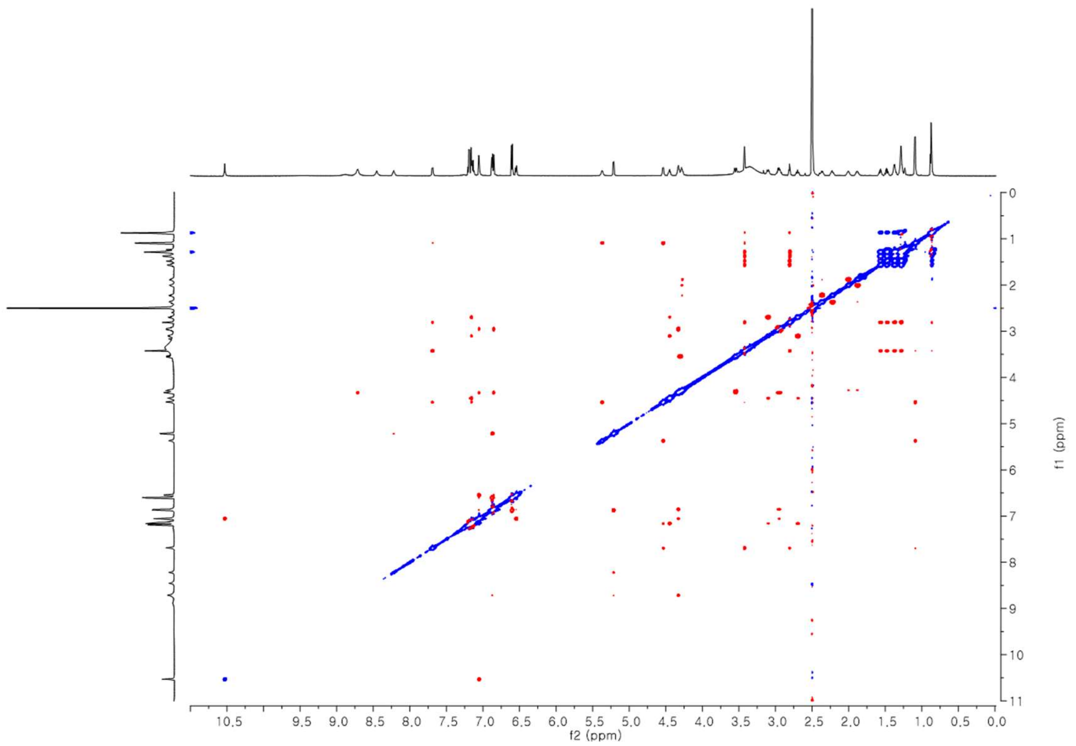

Figure S21. ROESY NMR spectrum of cystargamide D (**3**) at 700 MHz in DMSO-d_6.

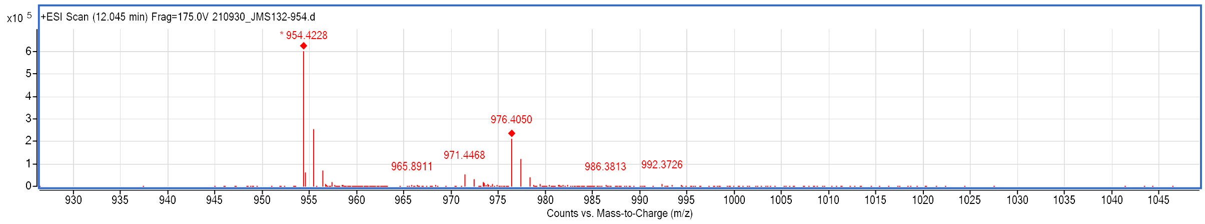

Figure S22. HR-ESI-MS data of cystargamide B (**1**).

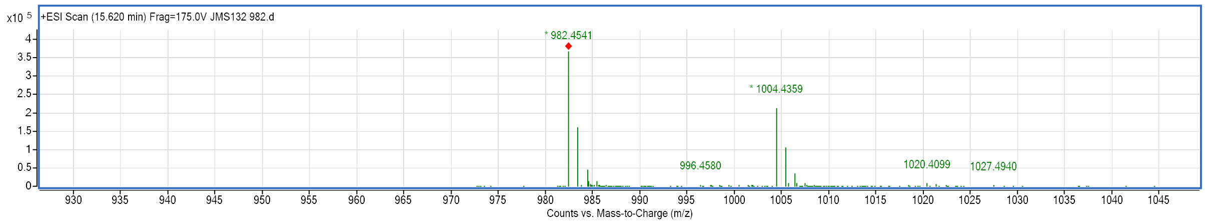

Figure S23. HR-ESI-MS data of cystargamide C (**2**).

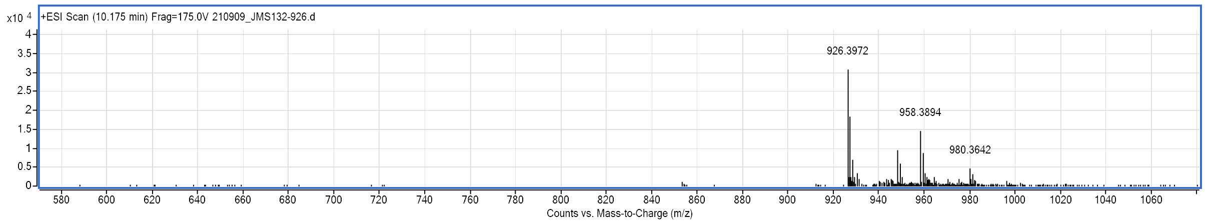

Figure S24. HR-ESI-MS data of cystargamide D (**3**).

(a) L-FDLA derivatives of the amino acid standards

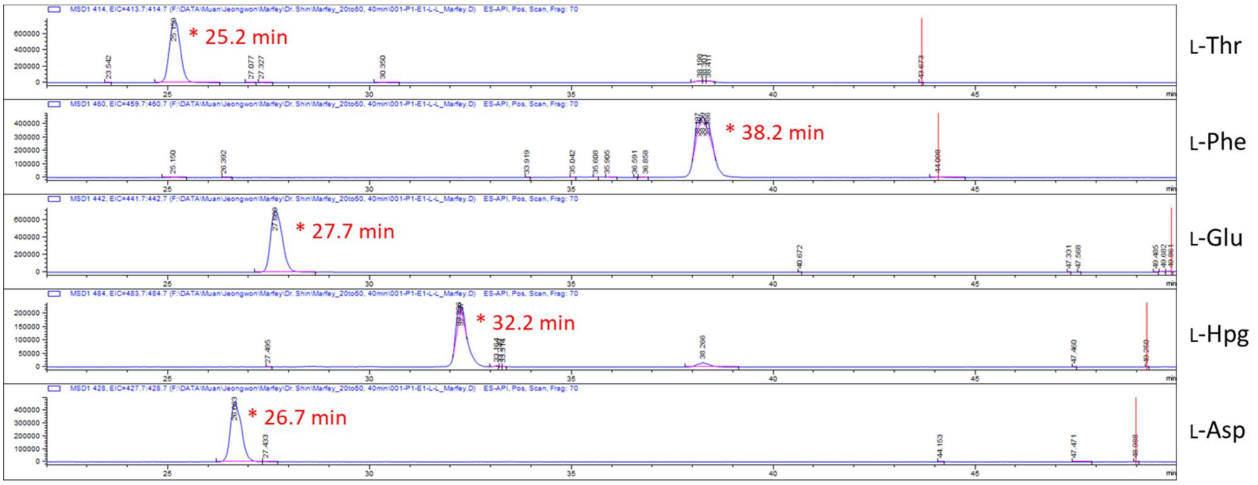

(b) D-FDLA derivatives of the amino acid standards

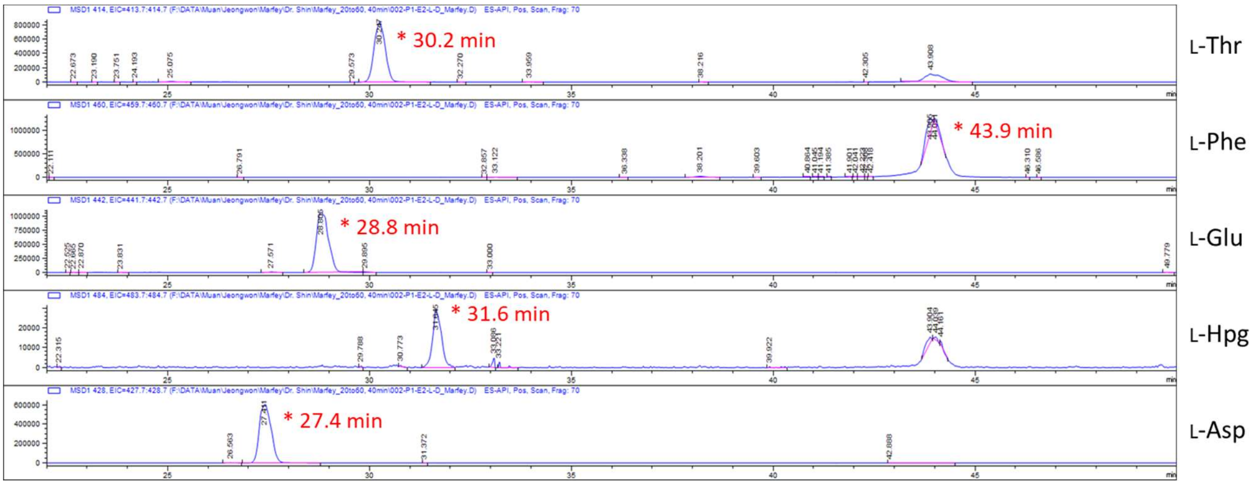

Figure S25. Extracted-ion chromatograms of L- and D-FDLA derivatives of the amino acid standards; L-Thr ($[M+H]^+$ = 414 *m/z*), L-Phe ($[M+H]^+$ = 460 *m/z*), L-Glu ($[M+H]^+$ = 442 *m/z*), L-Hpg ($[M+Na]^+$ = 484 *m/z*), and L-Asp ($[M+H]^+$ = 428 *m/z*).

(a) L-FDLA derivatized hydrolysate of cystargamide B (**1**)

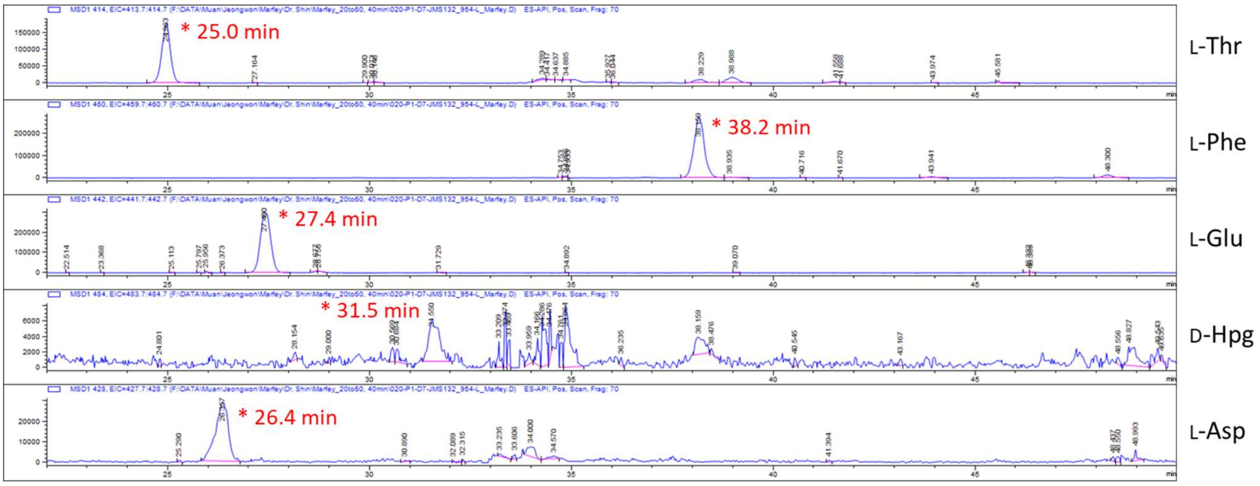

(b) D-FDLA derivatized hydrolysate of cystargamide B (**1**)

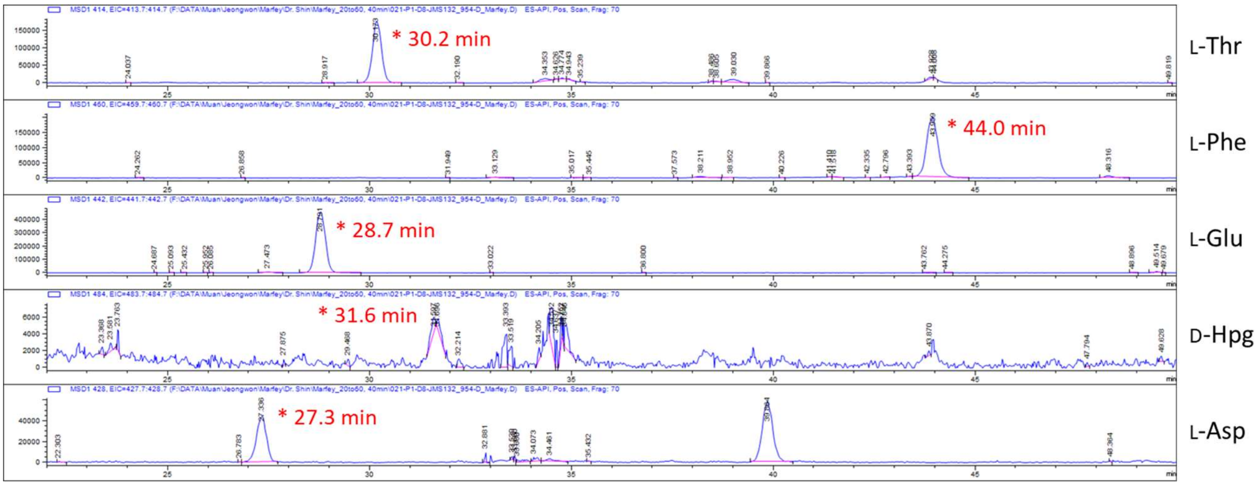

Figure S26. Extracted-ion chromatograms of L- and D-FDLA derivatized hydrolysate of cystargamide B (**1**).

Table S2. LC/MS analysis data of L- and D-FDLA derivatives of the amino acids.

amino acids	t_{RL}[b] (min)	t_{RD}[c](min)	elution order
L-threonine[a]	25.2	30.2	L → D
threonine from **1**	25.0	30.2	L → D
L-phenylalanin[a]	38.2	43.9	L → D
phenylalanin from **1**	38.2	44.0	L → D
L-glutamic acid[a]	27.7	28.8	L → D
glutamic acid from **1**	27.4	28.7	L → D
L-hydroxyphenylglycine[a]	32.2	31.6	D → L
hydroxyphenylglycine from **1**	31.5	31.6	L → D
L-aspartic acid[a]	26.7	27.4	L → D
aspartic acid from **1**	26.4	27.3	L → D

[a] *authentic amino acid standards*

[b]*Retention times of L-FDLA derivatives*

[c]*Retention times of D-FDLA derivatives*

Table S3. antiSMASH output table of *Streptomyces* sp. JMS132.

Region	Type	From	To	Most similar known cluster
Region 1	NRPS-like	178,374	219,279	diisonitrile antibiotic SF2768 (11% of genes show similarity)
Region 2	terpene	405,762	431,150	isorenieratene (100% of genes show similarity)
Region 3	T1PKS, lassopeptide	521,505	643,922	stambomycin A / stambomycin B / stambomycin C / stambomycin D (56% of genes show similarity)
Region 4	indole	808,442	829,569	5-isoprenylindole-3-carboxylate β-D-glycosyl ester (23% of genes show similarity)
Region 5	terpene	865,741	885,826	carotenoid (27% of genes show similarity)
Region 6	ectoine	1,976,303	1,986,701	ectoine (100% of genes show similarity)
Region 7	melanin	2,788,079	2,798,537	melanin (40% of genes show similarity)
Region 8	siderophore	2,888,910	2,899,410	desferrioxamin B / desferrioxamine E (83% of genes show similarity)
Region 9	other, NRPS	3,878,044	3,956,202	hormaomycin / hormaomycin A1 / hormaomycin A2 / hormaomycin A3 / hormaomycin A4 / hormaomycin A5 / hormaomycin A6 (95% of genes show similarity)
Region 10	terpene	4,972,979	4,992,523	albaflavenone (100% of genes show similarity)
Region 11	T2PKS	5,022,748	5,095,281	spore pigment (66% of genes show similarity)
Region 12	siderophore	5,540,999	5,552,032	
Region 13	arylpolyene, NRPS, NRPS-like, ladderane	5,674,398	5,759,301	WS9326 (92% of genes show similarity)
Region 14	RiPP-like	5,911,735	5,922,377	
Region 15	terpene	5,929,446	5,950,090	geosmin (100% of genes show similarity)
Region 16	NRPS, siderophore	6,064,133	6,124,748	lipopeptide 8D1-1 / lipopeptide 8D1-2 (20% of genes show similarity)
Region 17	terpene	6,411,532	6,437,250	hopene (92% of genes show similarity)

Region 18	hglE-KS, T3PKS	6,478,550	6,568,846	streptovaricin (19% of genes show similarity)
Region 19	RiPP-like	6,784,133	6,794,348	informatipeptin (28% of genes show similarity)
Region 20	NRPS-like, NRPS, terpene	7,228,086	7,288,243	thiocoraline (21% of genes show similarity)
Region 21	lassopeptide	7,354,622	7,377,329	kanamycin (2% of genes show similarity)
Region 22	lanthipeptide-class-I	7,492,935	7,516,681	
Region 23	lanthipeptide-class-I	7,617,040	7,642,226	
